# TICK: A Knowledge Processing Infrastructure for Cognitive Trust in Human–Robot Interaction

**DOI:** 10.1007/s12369-024-01206-1

**Published:** 2025-01-04

**Authors:** Mohammed Diab, Yiannis Demiris

**Affiliations:** Personal Robotics Laboratory, Imperial College London, South Kensington Campus, London, UK

**Keywords:** Trust infrastructure, Knowledge-driven approach, Human–robot interaction, Interaction memories

## Abstract

In order to assess trust within domains necessitating Human–Robot Interaction (HRI), such as social and assistive robotics, a multifaceted cognitive infrastructure is essential to facilitate shared knowledge among the participants. This knowledge encompasses information pertaining to the behaviour, beliefs, intentions, and situational awareness of the participants, including assessments of vulnerability, reliability, and risk. The developed knowledge takes the form of a domain-agnostic ontology with multiple layers, allowing for reusability across diverse HRI tasks, and resulting in actions that can be undertaken by a participant to establish trust with the other party. This research introduces the concept of Trust-Inferring Infrastructure for Cognitive Knowledge (TICK) for domains requiring HRI, comprising three key constructs: performance, process, and purpose. Subsequently, the proposed infrastructure is evaluated through real-world scenarios involving humans as trustors and robots as trustees. To validate TICK in these real scenarios, a multimodal sensory module has been integrated into the reasoning mechanism, enhancing the robots’ capabilities in understanding human intentions, perceiving the current situation, offering advice to humans, personalising their behaviour to build human trust, and evolving the robot’s trustworthiness based on its performance during interactions. Trust is automatically assessed based on proximity and adherence to the robots’ recommendations. Furthermore, to quantitatively assess the scalability and flexibility of the proposed approach, a series of experiments were conducted involving a kitchen domain handover task between humans and a table-top robotic arm in a lab setting, encompassing various types of objects and different scene contexts.

## Introduction

In recent years, robots have become increasingly common in numerous sectors and have fueled significant interest in HRI, including healthcare, manufacturing, and personal assistance. With this increased usage, it becomes essential to establish trust between humans and robots particularly when decisions made by Artificial Intelligence (AI) models can have significant real-world consequences that help to ensure their safe and effective interaction. This requires robots to perform specific joint tasks efficiently and achieve goals shared with humans at the best possible level. In order to increase the robots’ performance, traditional machine learning (ML) algorithms are widely used to enhance the robot’s vision, speech, and planning. However, one of the key challenges is to build an adaptive framework providing a common understanding of trust with participants in such HRI domains.

Traditionally, trust has been addressed through the use of machine learning algorithms [1], which are designed to learn patterns and make decisions based on training data. While these algorithms can be highly effective in many applications, they have limitations when it comes to explicitly representing and reasoning about trust. Despite the usefulness of these algorithms, they typically rely on learning patterns and relationships from large datasets and often produce “black box” models that are difficult either to be interpretable or to provide explainable AI. Moreover, they often struggle with issues such as overfitting, generalisation, interoperability, capturing uncertainty, and being transparent. On the other hand, the knowledge-driven approach can capture uncertainty and can produce transparent models that can be understood and easily explained by non-subject-matter experts. This can overcome the ML limitations by (a) representing knowledge of the sources and types of evidence that underpin decisions made by robots in a systematic and formal way, and (b) integrating experience-based knowledge learned by robots during interactions. This can facilitate more effective integration of multiple sources of knowledge and evidence. For example, it can integrate data from multiple sensors and modalities, as well as knowledge from multiple domains and sources. These advantages can enable more robust, transparent, interpretable and accurate decision-making, as well as enable the robots to adapt to new and evolving situations. Despite these advantages, there are still implementation challenges associated with knowledge-driven approaches. These challenges include the need to gather knowledge from domain experts and formalize it into a structured knowledge base, thereby increasing the complexity of the implementation process.

Knowledge representation formalisms such as ontologies provide a way to represent domain-specific knowledge. This involves creating a formal representation of the knowledge and reasoning methods to manipulate the knowledge. These formal representations enable the robot to reason about the domain-specific knowledge and make more informed decisions, leading to more reliable and trustworthy behaviour. For example, for HRI in a medical setting, knowledge representation can ensure that the robot follows medical guidelines and protocols as humans, leading to more reliable and accurate diagnosis and treatment. Similarly, in indoor settings, the use of knowledge representation can be beneficial in ensuring that the robot understands safety standards to prevent accidents and injuries. Furthermore, experience-based knowledge is also essential for establishing trust in HRI where the robots can learn from their past experiences and interactions with humans. By incorporating past interactions into their knowledge representation, robots can make better decisions about how to interact with humans, i.e., allowing them to better understand and respond to specific human needs and preferences, which is called Personalisation. For instance, a robot that has successfully completed a task before will be more likely to perform that task more effectively when including the personalisation aspect. This builds trust in the robot’s ability to perform the task and increases the likelihood that humans will trust and rely on the robot in the future. This can be widely applied in such domains including indoor settings where the robots can be trained to recognise the unique preferences and habits of an individual, enabling them to work more efficiently and effectively. Also, in the healthcare domain where robots provide tailored care to patients, taking into account their medical history, preferences, and needs.

Trust is a complex construct that involves the perception of the robot’s and human’s abilities, intentions, reliability, personalisation, vulnerability, safety, and others [2, 3]. Measuring trust in HRI requires a multidimensional approach that involves both subjective and objective measures [4, 5]. The former refers to an individual’s perception or feeling of trust towards a particular person, robot or entity. It is often based on the emotions and beliefs of a group of participants in a specific task. This is usually done through questions  [6–8], and [9] and can be shared later to let new participants learn from others’ past experiences. An example of the subjective measure of trust is the Robot Trust Scale (RTS) [10], which measures trust in social robots by asking participants to rate their agreement with statements related to the robot’s perceived competence, intention, and reliability. The RTS has been used to evaluate trust in various HRI scenarios, such as child-robot interactions and assistive robotics for the elderly  [4, 11]. However, subjective trust can lead to bias and prejudice, as participants’ judgments may be influenced by individual biases and prejudices [5]. To promote safe and effective HRIs, it is essential to understand the limitations of subjective trust and consider objective measures of robot performance. The latter is based on observable behaviour and refers to a more quantifiable and evidence-based approach to measuring trust. It typically involves gathering and analysing data related to specific behaviours, actions, and outcomes, then using this information to assess the level of trust of the person towards the robot either in an explicit or inferred manner [5]. The explicit manner can be done through straightforward questions. The inferred manner is more challenging and can be measured by observing a set of trust behaviours. Proximity is an example of the objective trust measurement that has been used to assess trust in human–robot interactions. This method involves how far the participants are from the robot during its movements executing the joint task which can provide insights into the level of attention and engagement they have with the robot. According to  [5], there is a prevailing tendency to rely more on subjective questionnaires than on objective measures when evaluating trust. However, the use of objective measures provides valuable insights into how individuals interact with robots, without relying solely on self-reported perceptions such as motivations or reasons. This approach allows for a more comprehensive understanding of trust and can lead to the development of more reliable and effective robots for diverse applications.

To measure objective trust, several ways can be exploited as presented in  [4]: (a) *Performance measures*: by analysing the performance of the human–robot team on a task. This can include completion time, accuracy, and efficiency. If the team performs well, it can be an indication of trust in the robot’s capabilities. (b) *Behavioural measures*: by analysing the number of times a person looks at the robot or how close they stand to it can also provide objective measures of trust. If a person feels comfortable enough to stand close to the robot or make eye contact, it can be a sign of increased trust. (c) *Self-assessment measures*: by analysing data about the robot’s performance in the past including its components such as vision and speech performance. These can be mathematically estimated based on the number of successful interactions among the entire ones. This can be useful if the robot has the ability to inform the participants about the trustworthiness level in previous tasks beforehand, which can increase the objective measures of trust. (d) *Physiological measures*: by analysing physiological signals such as heart rate variability, skin conductance, and eye tracking which can provide objective measures of trust. For example, if a person’s heart rate variability increases or their skin conductance decreases when working with a robot, it can be a sign of increased trust.

In this article, our interest is designing a modular knowledge-driven trust infrastructure, called TICK that can be readily adapted to measure objective trust mostly in an inference manner that suits new scenarios. We will focus on the *Performance, Behavioural* and *Self-assessment* measures. Irrespective of the target scenario, we assert that there are core components that can be reused across multiple instantiations of the trust infrastructure: perceiving user identity, understanding human state and actions, constructing memories of interactions, evolving the robot’s trustworthiness while interacting, reasoning about the context and trust factors, selecting actions that are optimal for this user and task, and then adapting the robot behaviour to calibrate the human trust. The main contributions and novelty of this work are proposing a knowledge-driven framework TICK that contains definitions of several factors that influence trust measurements and ground them to evaluate trust that is used later to personalise the robot behaviours towards the participants. The contributions are:

**Fig. 1:**
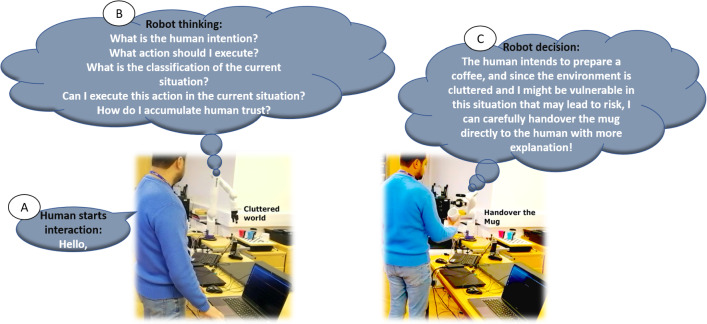
Prototypical example task: object handover in kitchen domain. The Kinova-gen3 7-DoF robot with a Robotiq 2F-85 two-finger gripper assists humans with coffee preparation tasks. **a** The human starts the interaction with the robot by saying hello. **b** The robot starts asking some questions aiming to understand the scene and human intention. **c** The robot makes a decision about the suitable behaviour to be executed with the human based on the answer to the previous questions aiming to accumulate human trust

- Introduction of an ontological trust infrastructure that includes a large number of factors that influence trust compared to the state-of-the-art. This infrastructure contains knowledge-agnostic levels aiming at generality, shareability between multi-agents, reusability, and adaptability in different tasks.
- The scaled interaction memory in order to store the interactions and the necessary data to be asserted to the KB. An interaction memory is used to store context-based episodes, which refers to specific states of environmental entities in the past, including humans and robots.
- Integration of assistant modules, including multi-modal perception and planning, to automatically perceive the environment states and build domain-specific knowledge by integrating different data-based sources to manage large amounts of interactions. Moreover, to increase the robot’s ability to work in real-world environments that include cluttered and dynamic objects.
- A heterogeneous inference mechanism aiming at better inferring trust signals of humans while the interaction between humans and robots.

The objective of this work is to enable a robot to personalise its behaviour based on the level of trust inferred from a human. To achieve this, we propose handover scenarios that can be widely used in various situations and domains. We demonstrate the effectiveness of our approach by applying the handover task in the kitchen domain, where the human is viewed as a trustor and the robot as a trustee. In Fig. 1, we present a scenario where a human and a Kinova Gen3 robot share the handover task of preparing coffee in a cluttered environment. We propose two different options for the robot’s behaviour: a direct handover, which is the optimal choice and preferable if the human trusts the robot to execute it competently, and an indirect handover, where the robot picks up the object but places it on a table in front of the human if the human does not trust the robot to execute the task. These options are intended to personalise the robot’s behaviour towards a specific user based on the level of trust inferred from that user. Our proposal represents a significant step towards enabling robots to adapt their behaviour to individual users, enhancing the overall human–robot interaction experience.

The paper follows a structured framework, beginning with the theoretical background in Sect. 3, followed by the case study and scenario descriptions, and the implementation details in Sect. 4. Section 5 presents the experimental results, while Sect. 6 discusses the approach and results. Finally, Sect. 7 concludes the paper and outlines future directions.

## Related Work

### Trust Definition

Trust is a fundamental concept across various fields of research, including psychology [12], sociology [13], economics [14], and management of the Internet of Things (IoT) [15]. Trust has been explored from diverse perspectives in the literature, resulting in a variety of definitions. For instance, Mayer et al. [16] defines trust as the willingness of individuals or groups to rely on and expose themselves to the actions or decisions of others. Gambetta et al [17] defines trust as an expectation of positive outcomes based on beliefs about the intentions, competence, and dependability of others. Castelfranchi and Falcone  [18] defines trust as a social exchange mechanism that involves the transfer of resources, information, or power between parties. Furthermore, the cognitive or emotional state model of trust is emphasised  [19], which affects attitudes, behaviours, and relationships between individuals or organizations.

Within the domain of HRI, Lee and See [20, 21] propose that the vulnerability of either the trustor or the trustee significantly affects the level of trust. Castelfranchi and Falcone [18] and Luhmann [22] also emphasise the importance of uncertainty and risk when evaluating trust. Additionally, a model proposed in [19, 23] highlights the role of belief in the interaction between the trustor and trustee, particularly in physical interaction. Despite significant progress in clarifying the essential facets of trust, the term remains semantically overloaded, lacking a widely agreed upon, and conceptually clear definition, with critical implications for facilitating cooperation, reducing uncertainty, and enhancing social well-being [18].

### Factors Influence Trust in HRI

Trust is a notion that is increasingly being used in HRI applications. Several studies have been conducted to explore the various factors that affect trust in these interactions. One of the most significant factors that influence trust in human–robot interactions is reliability. Several studies have shown that users tend to trust robots more when they are reliable and consistent in their performance. For example, in  [3, 24], participants were more likely to trust a robot that consistently performed a task correctly, even if the robot appeared less human-like in its movements. Another factor considered that affects trust is the robot’s appearance and behaviour. The robot’s appearance can play a significant role in establishing trust, with more human-like robots often being trusted more than less human-like robots [25, 26]. Additionally, the robot’s behaviour, including its communication style, exemplified by the speed of interaction, ranging from rapid exchanges to slower interactions observed in handover tasks, and social cues, can influence how much users trust it. For instance, in  [20], participants were more likely to trust a robot that displayed social cues such as nodding and maintaining eye contact during a conversation. Transparency also affects trust in human–robot interactions. When robots are transparent about their intentions and decision-making processes, users tend to trust them more. This can be achieved through various means, such as providing users with explanations of the robot’s actions or allowing them to see the robot’s internal processes. For example, in  [27] and  [28] users were more likely to trust a robot that provided them with explanations of its actions. The context of the interaction also plays a role in influencing trust. In situations where users perceive a higher risk, the users tend to trust robots more if they perceive them as capable and reliable. In contrast, in less risky situations, users may be more willing to take risks and trust robots with less reliability or lower levels of transparency [29, 30]. The user’s prior experience with robots can also influence how much they trust them. If users have had positive experiences with robots in the past, they are more likely to trust them in future interactions. Conversely, negative experiences can erode trust in robots [31].

While multiple factors related to trust in human–robot interaction have been explored, certain important aspects such as robot vulnerability and human intention in real-world settings remain inadequately addressed. Additionally, there is a need to develop an adaptable tool that encompasses most of these factors and can be exploited to assess trust in online settings.

### Trust Ontologies in HRI

In recent years, there has been growing interest in using ontologies for trust in HRI. Using ontologies for trust in HRI can provide a formal and structured way to represent and reason about trust relationships between humans and robots. These ontologies can also facilitate the development of intelligent systems that are capable of adapting to different contexts and individual preferences. One of the early works in this area is the Trust Ontology proposed in  [32] which formalises the concept of trust by defining and linking other concepts such as beliefs, expectations, transparency, reliability, and competence. Another ontology, “The Reference Ontology of Trust”, is proposed in [33] which formalises the concept of trust in robots by defining its different facets, such as reliability, belief, and intention. Moreover, the Ontology of trust which is proposed in  [34] represents trust relationships between humans and robots using a set of axioms and rules. It considers different factors that influence trust, such as the robot’s appearance, behaviour, and context of use. In semantic web applications, the trust networks ontology on the semantic web [35] is proposed which introduces an ontology used to model trust factors such as safety in the connections between humans and AI systems through the semantic web network.

All of these ontologies focus on providing a very abstract model that can be used in several domains. Our focus is developing more comprehensive and adaptable ontologies that are validated empirically and can be readily applied across different types of robots and scenarios. Our ontology aims at (a) a nuanced understanding of the complexity of trust in HRI with more detailed of how trust operates in HRI rather than focusing on specific aspects of trust, such as emotional trust. (b) Validation of our ontology in real scenarios not just theoretical models to ensure that the ontology accurately captures the factors that influence trust in HRI. (c) A context-independent ontology that can be adaptable to different scenarios or environments. This can increase its usefulness in real-world applications where the robot needs to adapt to different contexts and users. (d) A standardised version of trust ontology in HRI using upper-level ontological foundations, which can facilitate comparison and integration of different models.

***Upper-level ontological foundation:***

The upper-level foundations focus on providing upper-level models, i.e., concepts and relations in a generic way aiming to be shared among different tasks and domains. SUMO (Suggested Upper Merged Ontology) [36] is the ontology that provides common vocabularies that are widely used in the autonomous robotics domain. It has been used in robotics frameworks such as [37]. Unified Foundational Ontology (UFO)  [38] aims to unify different upper-level ontologies and provide a common framework for knowledge representation. It includes concepts such as entities, events, qualities, and roles, and it provides formal semantics for representing different types of knowledge. DOLCE (Descriptive Ontology for Linguistic and Cognitive Engineering) [39] is the ontology that provides common vocabulary with relations between these concepts that are widely used in the cognitive HRI domain. It has been used in the robotics framework like  [40]. DOLCE-Ultralite (DUL) is a simplified version of the DOLCE ontology that expands its coverage to include the Descriptions and Situations framework. It simplifies some modal axioms of DOLCE to make it more lightweight. DUL has been the inspiration for the creation of some core ontology design patterns, and has been adopted in numerous ontology projects worldwide.

Compared to other upper-level ontologies, the DUL ontology is more tailored to the representation of concepts related to human cognition, which makes it a good fit for modelling trust in HRI. It provides a clear and comprehensive framework for representing concepts related to trust, such as beliefs, desires, intentions, and emotions, which are essential for modelling trust in HRI. Moreover, it is designed to be modular and extensible, which means that it can be easily extended to include new concepts and relations that are specific to the HRI domain.

## The Proposed Architecture

### Overview

**Fig. 2:**
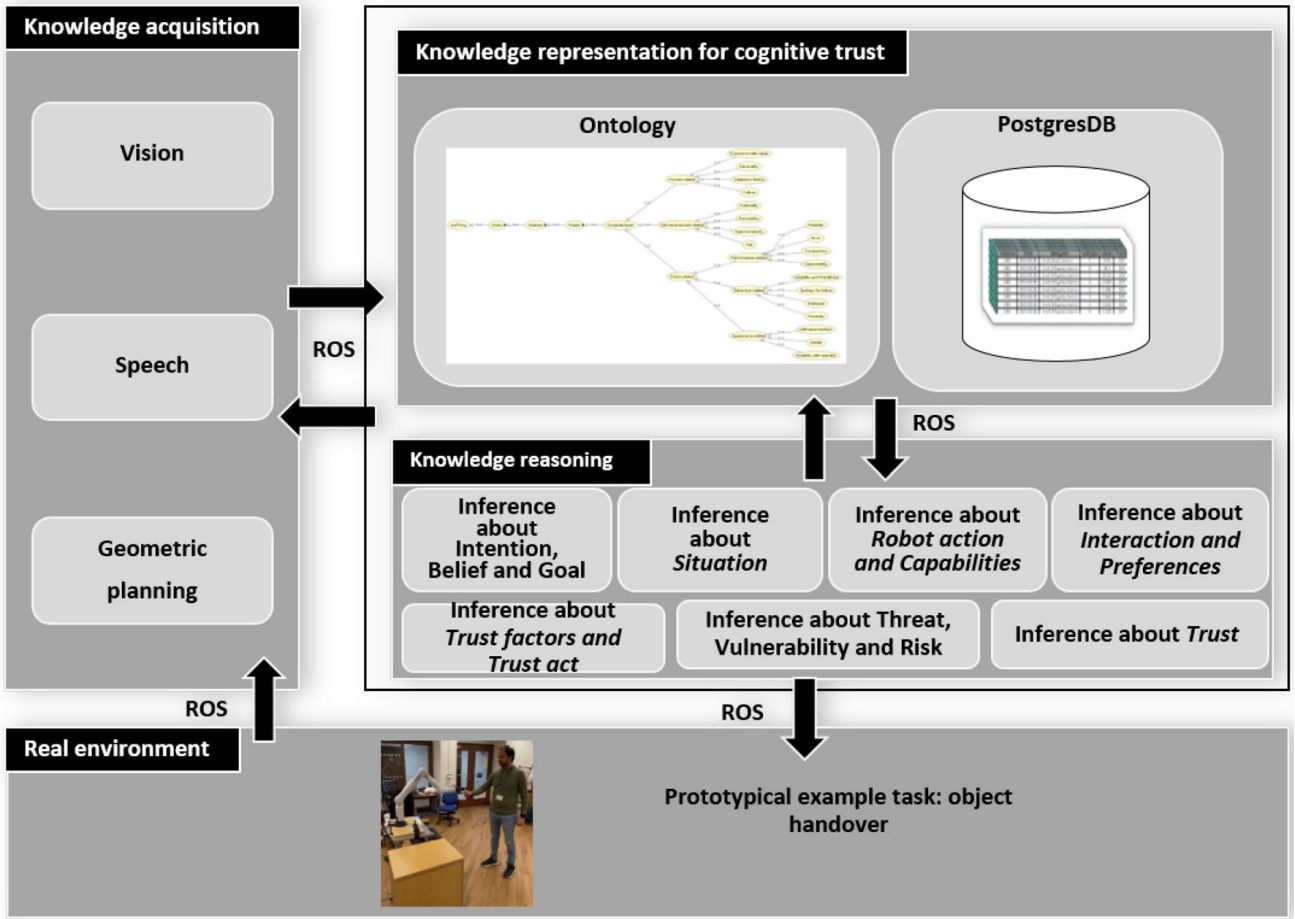
The TICK knowledge-based reasoning framework contains three main layers. (1) The knowledge acquisition layer contains **a** *the vision* to identify the human behaviour and environmental entities including their locations, **b** *the speech recognition system* to generate informative dialogues between humans and robots while interacting, **c** *the motion planner* to generate the collision-free trajectories to be executed by the robot, as well as evaluate geometrically the actions’ feasibility. (2) The knowledge representation layer contains **a** *the ontology* (static knowledge) which contains the abstract description of the trust and its components such as reliability and capability and situation awareness as well as the relations between the concepts which are used for describing the domain. **b** *the PostgreSQL* (dynamic knowledge) which contains the dynamic data expressing the context (i.e., the environmental entities). This data has been acquired by the knowledge acquisition layer and automatically asserted to the PostgreSQL DB. (3) The knowledge reasoning layer provides heterogeneous reasoned information related to the human goal, belief, intention, situation awareness, and the propitiate robot actions based on the current situation, vulnerability, and risk. Ultimately, a real scenario has been designed to execute the handover task in any domain (in this scenario, the handover task has been tested in the kitchen domain)

The TICK framework, as shown in Fig. 2, comprises of various core components which are essential for trust inference. These components include knowledge acquisition, knowledge representation, and knowledge reasoning. Knowledge acquisition involves collecting perceptual features of the environment entities through multi-modal sensory integration that enables vocal and visual interactivity. Knowledge representation provides an adaptable knowledge base about trust and its components for robots to use in reasoning tasks. TICK follows the cognitive-based engineering upper-level foundation concepts of DUL to standardise the modeling of knowledge in the human–robot interaction domain. This facilitates information exchange and increases interoperability and shareability of knowledge. Knowledge reasoning is responsible for providing an inference mechanism about trust and its components aiming at increasing the robot’s smartness enhancement and situation awareness. Explicitly, we develop an inference mechanism for the robot to reason about the human’s intention, the robot’s capability, and the best actions to be executed. Moreover, we develop a reasoning mechanism about the current scene, including the entities’ states, the vulnerability and risk levels, and the robot’s reliability in the current situation. The components are semantically linked, forming a knowledge graph that facilitates trust inference in the TICK framework.

The trust measurements in TICK are influenced by various factors, including risk assessment, robot reliability, explainability, human intention, proximity, following recommendations, and personalisation. These factors are acquired and incorporated into the ontological knowledge-based representation model using multi-modal sensory sources. For instance, the depth information obtained from the robot’s visual sensor is used to detect the distance between the robot and the human, thereby enabling the robot to monitor and comprehend the user’s reactions to its actions. This facilitates the robot’s understanding of trust in a standardised manner close to humans, mitigating the possibility of task execution failures and associated accidents. Based on the human’s perceived reaction and the inferred level of trust, the robot records the interaction episode and adjusts its subsequent behaviour accordingly. Additionally, the robot self-estimates its trustworthiness level concerning the performance of its components after each iteration and clarifies it to the participants during the interactions. The trust measurements in TICK are influenced by various factors, including risk assessment, robot reliability, explainability, human intention, proximity, following recommendations, and personalisation. These factors are acquired and incorporated into the ontological knowledge-based representation model using multi-modal sensory sources. For instance, the depth information obtained from the robot’s visual sensor is used to detect the distance between the robot and the human, thereby enabling the robot to monitor and comprehend the user’s reactions to its actions. This facilitates the robot’s understanding of trust in a standardised manner close to humans, mitigating the possibility of task execution failures and associated accidents. Based on the human’s perceived reaction and the inferred level of trust, the robot records the interaction episode and adjusts its subsequent behaviour accordingly. Additionally, the robot self-estimates its trustworthiness level concerning the performance of its components after each iteration and clarifies it to the participants during the interactions.

### Knowledge Acquisition Layer

A multi-modal sensory module, using vision and speech, has been proposed to identify the human, physical objects, and their properties. The identification of the human involves recognising the person’s physical features and linking this information to their identity. Additionally, the identification of physical objects and their properties provides information about the environment and can help in determining the purpose of the human actions. This step is critical in providing a context for analysing the human actions and inferring trust. The identification of the human involves recognising the person’s physical features and linking this information to their identity. Once these elements have been identified, the next step is to analyse the human actions and infer their level of trust. This involves proximity and language, and verbal cues.

### Knowledge Representation Layer: Static Knowledge

In TICK, an ontological-based representation model is proposed to provide a taxonomy of static knowledge which is then used to understand the meaning of collected data from the knowledge acquisition layer. Static knowledge refers to general knowledge about the facts and concepts that can be independent of an agent’s specific experience. This type of knowledge requires appropriate conceptualisation in order to be readily adapted to different tasks. The conceptualisation represents an abstract model of entities in a specific domain, achieved by defining their relevant concepts along with their relations. We provide an overview of knowledge structure and how to connect them to the real world. The concepts formalised in the ontology are highlighted using Roman font style, and the reader is encouraged to refer to the dictionary in the Appendix to understand the meaning of the ontological concepts. The proposed taxonomy provides a structured approach for knowledge representation, which can facilitate efficient reasoning and decision making in autonomous systems.

**Fig. 3:**
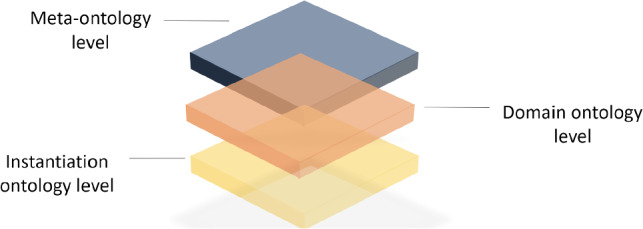
The knowledge structure contains 3-level ontologies: **a** Meta-ontology level: Upper-level ontology that has common and general concepts to be used. **b** Domain ontology level: Ontology that describes a specific domain such as an object handover in a kitchen domain. **c** Instantiation ontology level: Ontology for individuals, or instantiation knowledge

**Taxonomy structure:** We propose a hierarchical taxonomy structure of ontologies to facilitate the robot’s understanding of the concept of trust and establish a shared conceptual foundation with humans prior to interacting, to promote mutual trust. The taxonomy structure consists of three levels of ontology hierarchy: metaontology, domain ontology, and instantiation ontology levels, as illustrated in Fig. 3. The metaontology level represents generic information such as the concept of Trust, while the domain ontology level contains domain-specific knowledge related to a particular task, such as the object-handover task. Finally, the instantiation ontology level stores information about specific objects such as kitchen objects and their features. The object-oriented and frame-based ontology language allows the metaontology layer to provide a template for the domain ontology level, while the ontology instance layer can be defined as an individual frame. Bidirectional reasoning is used to transfer information of ontological classes, properties, and instances within the same knowledge layer, while unidirectional reasoning is used to relate multiple knowledge classes of different layers.

In real-world environments, HRI tasks often require knowledge of general tasks, behaviours, and manipulations. However, conventional knowledge frameworks are typically task-specific, limiting their applicability to broader contexts. To address this issue, a metaontology has been formalised based on the DUL upper-level foundation, with the aim of generalising the approach and making it widely available in robotic systems. The choice of DUL is based on several reasons: (a) it has a clear cognition engineering and employs natural language processing techniques that incorporate human-like commonsense knowledge. This enables the categorization of abstract models and the establishment of connections between them based on intuitive understanding, (b) it has a manageable number of concepts with clear descriptions that facilitate the ontology alignment process, and (c) it is an open-source ontology. By leveraging DUL as the basis for metaontology, robotic systems can better leverage generalised knowledge frameworks for a range of human–robot interaction tasks.

### Modeling Trust and Its Factors: Metaontology Level Modelling

There are two main questions we can answer using this ontology: what is Trust? and what are the factors affecting Trust? Based on the literature, we have found the most suitable definition for trust to be the one proposed by  [20], which states that trust is *“the attitude that the agent will help achieve an individual’s goals in a situation characterized by uncertainty and vulnerability”*.^1^ Although this definition is broad and captures most scenarios in the HRI literature, we have considered it from a general perspective that trust is a dynamic element affected by where, when, and how the interaction happens. In our case study, we have added more clarifications to the definition, including the explicit meaning of uncertainty (i.e., related to which participant or the environment) and other factors that affect trust evaluation. We believe factors such as risk, reliability, explainability, as well as the personalisation aspect of how the robot can adapt its action based on human behaviour, can impact the evaluation of trust. Additionally, human intention, goal, and belief play an essential role. Therefore, we have added minor clarifications to the definition. For this work, we adopt a fuller definition, which states that trust is *“the process that represents the state of the entities, includes agents sharing a task with individuals to achieve their goals, in a series of events that can be calibrated over time in a situation characterised by uncertainty related to human, robot or environment”*.^2^

We have conducted a study to identify potential factors that contribute to the development of trust in the human–robot domain. Our analysis, based on the work of [41], has revealed that these factors include prior experience, attentional capacity, expertise, competency, personality, attitudes, the propensity to trust, self-confidence, false alarm, failure rate, automation, anthropomorphism, predictability, proximity, robot personality, multitasking, workload, task load, culture, shared mental models, and situation awareness.

Furthermore, we argue that reliability, vulnerability, risk, and personalisation, are also crucial for the development of trust. The main challenge from an ontological perspective is to categorise and link these factors at the metaontology level. In our trust model, we have modeled all the aforementioned factors at the metaontology level and have utilised them in our case study.

#### Trust Model

The proposed definition of Trust can be viewed as a relational construct encompassing multiple interconnected concepts. Each pair of concepts is linked together through a specific relation. For example, trust is associated with an agent, denoted as Agent, representing an abstract entity that can be either human or robot. This connection is expressed by the relation *includesAgent*.

Trust is represented as a tuple consisting of ten elements: $\langle$ a, t, p, s, r, re, si, v, i, ex $\rangle$. Each element represents a distinct aspect of trust, as follows:

- a: Agent, indicating the number of participants collaborating, including at least one Trustor and one Trustee.
- t: Task, identifying the specific task or subtask in which the agents are involved.
- p: Interaction, describing the manner in which the two agents interact with each other.
- s: Social attribute, capturing the factors that can influence trust.
- r: Risk assessment, determining the level of risk and its impact on the evaluation of trust.
- re: Reliability, inferring the capability of an agent in a given situation.
- si: Situation, characterizing the contextual setting and facilitating a shared understanding among the agents.
- v: Vulnerability, identifying the susceptibility of agents in a particular situation or task.
- i: Intention, elucidating the intended actions or goals of a human agent.
- ex: Explainability, discerning the optimal mode of interaction between agents collaborating on the same task.

The integration of the aforementioned elements has been employed to establish a formal representation of trust within the TICK ontology, employing the framework of First Order Logic (FOL) [42] .^3^

$$ Trust(t) \equiv dul.Event(e) \wedge \exists y,z,t,p,s,r,re,si,v,i,ex$$ $$(y \ne z)$$

$$ dul.Agent (y) \wedge dul.includesAgent (e,y) \wedge $$

$$ dul.Agent (z) \wedge dul.includesAgent (e,z) \wedge $$

$$ dul.Task (t) \wedge dul.hasConstituent (e,z) \wedge $$

$$ dul.Interaction (p) \wedge dependsOn(e,p) \wedge $$

$$ dul.SocialAttribute (s) \wedge hasFactor(t,s) \wedge $$

$$ Risk (r) \wedge isAffectedBy(t,r) \wedge $$

$$ Reliability (re) \wedge isAffectedBy(t,re) \wedge $$

$$ Situation (si) \wedge isAffectedBy(t,si) \wedge $$

$$ Vulnerability (v) \wedge isAffectedBy(t,v) \wedge $$

$$ Intention (i) \wedge isAffectedBy(t,i) \wedge $$

$$ Explainability (ex) \wedge isAffectedBy(t,ex) $$

$$ \forall Trust (t) \rightarrow isResultedIn a (Action) \wedge a \in (trustAct) or (disTrustAct) $$

The definition of Trust can be understood as follows: trust is characterised as an event (e) wherein a minimum of two agents are involved, identified as the trustor (y) and trustee (z), engaging in a specific task or sub-task (t) that necessitates their interaction (p). The dynamics of trust are influenced by social attributes (s), which encompass factors that impact trust, such as the inference of human intention (i), the agent’s capability in a given situation (re), vulnerability (v), and risk (r). The outcome of trust manifests as either an act of trust or an act of distrust, resulting in subsequent actions.

The concept of Social attribute is a construct inherited from the DUL upper-level ontology. It is specifically defined as “Any Region in a dimensional space that is utilised to represent a particular characteristic of a Social object, such as judgment values, social scalars, or statistical attributes over a collection of entities” [39]. In our framework, we establish three primary sub-classes within the Social attribute concept: human-related, robot-related, and task and environment-related. The human-related sub-class pertains to the model associated with humans, encompassing factors such as personality traits, cultural background, prior experience with robots, subjective feelings, and similar attributes. It delves into understanding the human perspective and its influence on social interactions. The robot-related sub-class pertains to the model associated with robots, considering behavioural aspects such as proximity to humans, performance-related attributes like transparency and error rates, and appearance-related attributes such as anthropomorphism and resemblance to other agents. This sub-class delves into the characteristics and qualities of robots that can impact social dynamics. The task and environment-related sub-class pertains to the model associated with the task and the environment in which the agents operate. It encompasses factors such as the collaborative aspects among multiple agents, constraints imposed by the task, and the overall environmental context. This sub-class captures the contextual factors that shape social interactions in a given scenario. By delineating these sub-classes, we aim to comprehensively capture the various dimensions of social attributes and their relevance within our framework.

#### Intention Model

We propose a definition of Intention specifically tailored for shared tasks, which can be described as follows: “Intention is a representation of how to accomplish a goal, as defined by the task instructions, within a specific task, based on the trustor’s belief towards the trustee.”

The intention is modeled as a tuple, denoted as $\langle$ b, g $\rangle$, where b represents the trustor’s belief towards the trustee and g represents the goal of the trustor, indicating the intended action to be undertaken by either the trustor or the trustee. The formal representation of intention is expressed in FOL as follows:

$$ \text {Intention}(i) \equiv \text {dul.InformationRealization}(ir) \wedge \exists x, y, b, g $$

$$ \text {dul.Agent}(x) \wedge \text {belongsTo}(i, x) \wedge $$

$$ \text {dul.Agent}(y) \wedge \text {towards}(i, y) \wedge $$

$$ \text {Belief}(b) \wedge \text {dul.hasPart}(i,b) \wedge $$

$$ \text {dul.Goal}(g) \wedge \text {dul.hasPart}(i,g) \wedge $$

$$ \forall y (\text {dul.Agent}(y) \wedge \text {hasBelief}(y,b) \wedge \text {hasGoal}(y,g) \rightarrow \text {hasIntention}(y, i))$$

The proposed definition of Intention can be interpreted as follows: intention (i) is a form of information that belongs to an agent (x) in the role of a trustor towards another agent (y) acting as a trustee. The trustor holds a belief (b) regarding what the trustee is capable of accomplishing, which is manifested by the defined goal (g).

#### Threat, Vulnerability and Risk Model

We present a proposed definition for the concept of Threat as follows: “A threat is an event that encompasses objects capable of causing harm or posing danger, thereby potentially rendering an agent vulnerable.” To illustrate this concept within the context of the TICK framework, an alarm indicating low battery levels in a robot would be considered a threat event. Similarly, a robot operating in a cluttered environment can also be perceived as a threat. The factors influencing a threat event can be represented as a tuple, denoted as $\langle \text {s}_o, \text {s}_a \rangle$, where s$_o$ signifies the state of physical objects within the environment, and $\text {s}_a$ represents the state of the agent.

The formal modeling of the threat concept within the TICK framework employs FOL and is expressed as:

$$ Threat(th) \equiv dul.eventType(et) \wedge \exists x, d, v $$

$$ dul.Agent (x) \wedge dul.hasPart(th, x) \wedge $$

$$ dul.Object (o) \wedge dul.hasPart(th, o) \wedge $$

$$ \forall th (dul.Agent(x) \wedge satisfies(th, d) \rightarrow leadsTo (th, v)) $$

In the above formula, the description of the threat event can be interpreted as follows: the states of either the agent (x) or the object (o) can give rise to a situation wherein the agent becomes vulnerable. The description (d) represents the contextual details of the threat event.

Vulnerability in the TICK is defined as a characteristic of an agent’s performance that indicates its weakness, encompassing both its components and the communication between them, which can be exploited by a threat event. For instance, a robot may be vulnerable to executing a lengthy task when its battery is in an extreme state, or vulnerable to object manipulation in a cluttered environment.

The factors influencing vulnerability can be represented as a tuple $\langle re, comp, exp \rangle$, where *re* denotes the reliability of the agent in executing a specific task or action, *comp* represents the components of the agent (such as the battery for robots or hands for humans), and *exp* reflects the agent’s prior knowledge or experience regarding its performance in executing the current task under the present circumstances. This experience can be regarded as the agent’s trustworthiness towards its own performance. Formally, vulnerability (*v*) can be defined in FOL as follows:

$$ Vulnerability(v) \equiv dul.Quality(q) \wedge \exists x, re, p, comp, exp, r$$

$$ dul.Agent(x) \wedge dul.hasPart(th, x) \wedge $$

$$ Reliability(re) \wedge dependsOn(v, re) \wedge $$

$$ Component(comp) \wedge isCausedBy(v, comp) \wedge $$

$$ Experience(exp) \wedge isReferenceOfInformationRealizedBy(v, exp) \wedge $$

$$ \forall v (dul.Agent(x) \wedge satisfies(r, d) \rightarrow leadsTo (v, r) \wedge hasEffect(v, p) ) $$ ;

where *r* refers to risk, and *p* represents the interaction.

Risk in the TICK is defined as a probabilistic value that has the potential to result in loss or harm due to the exploitation of vulnerability by a threat event. The assessment of risk takes into account both the threat event and the vulnerability of an agent. For instance, a robot can pose a risk if its speed is excessively high while collaborating with a human in a confined space. The factors influencing risk can be represented as a tuple $\langle$s, a, v$\rangle$, where *s* represents the current situation, *a* denotes the action being performed (such as picking or placing), and *v* represents the vulnerability. Formally, risk (*r*) is modeled in FOL as follows:

$$ Risk(r) \equiv dul.event(e) \wedge \exists so, ral, rn $$

$$ Source(so) \wedge hasFactor(r, so) \wedge $$

$$ RiskAssessmentLevel(ral) \wedge hasValue(r, ral) \wedge $$

$$ RiskNarrative(rn) \wedge dul.satisfies(r, rn) \wedge $$

$$ Vulnerability(v) \wedge isAffectedBy(r, v) \wedge $$

$$ \forall r (dul.Agent(x) \wedge dul.object(ob) \wedge \wedge satisfies(r, rn) \rightarrow hasEventType(e, r)) $$

This definition incorporates the concepts of event (*e*), source (*so*), risk assessment level (*ral*), risk narrative (*rn*), vulnerability (*v*), and their relationships. It indicates that risk assessment involves identifying the sources of risk, assigning a risk assessment level, and describing the risk narrative. Furthermore, risk is influenced by vulnerability, and in the presence of a specific risk narrative satisfaction, the event type is associated with the risk.

**Fig. 4:**
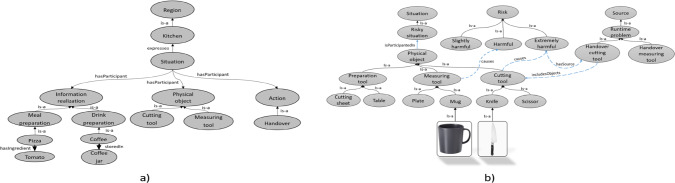
**a** The domain knowledge ontology. **b** The interpretation of the risky situation in the kitchen domain. The risk has been linked to the physical objects participating in the risky situation. The risk has been categorised into three main categories, slightly harmful, harmful, and extremely harmful. Besides the threat and vulnerability information and depending on the source of risk and the actual physical objects participating in the current situation, the risk is automatically mapped to one of the three categories

#### Situation Model

In TICK, Situation refers to relational contexts encompassing a set of events involving agents (e.g., humans and robots) and physical objects. These situations enable participant robots to create semantic descriptions of the relational context. In the context of TICK, a situation can be represented as a tuple $\langle a, o, e\rangle$, where *a* represents the action executed by both the human and the robot, *o* denotes the set of physical objects involved, and *e* represents the set of events that occurred. The definition explicitly follows the DUL (see the Appendix A.1) definition of the situation and introduces subcategories such as threat situations, vulnerable situations, and risky situations. These subcategories are classified based on the narrative descriptions that satisfy the threat, vulnerable, and risky criteria (the description is provided by the user based on the domain). Formally, the situation(s) is represented in FOL as:

$$ Situation(s) \equiv dul.situation(s) \wedge \exists o, a, e, d$$

$$ dul.Physical\ object(o) \wedge hasParticipant(s, o) \wedge $$

$$ Action(a) \wedge hasParticipant(s, a) \wedge $$

$$ dul.Event(s) \wedge hasParticipant(s, e) \wedge $$

$$ dul.Description(d) \wedge satisfies(s, d) \wedge $$

$$ \forall s (situation(s) \wedge satisfies(s, rn) \rightarrow hasSituationType(s, r)) $$

where “d” refers to the Description concept.

This definition asserts that a situation (s) follows the same definition as specified in DUL, wherein physical objects (o) including agents participate in an event (e), and the description of the situation should satisfy one of the narrative subclasses. These narrative subclasses are classified as threat narrative (tn), vulnerable narrative (vn), and risky narrative (rn).

#### Interaction

Interaction plays a significant role in evaluating trust within the field of HRI. In accordance with the standardised definition proposed in  [43], interaction refers to the alteration of the qualities and relationships of objects/agents involved in an event. This definition is adopted because it encompasses the behaviour of both the trustor and the trustee, as well as their reactions based on each other’s preferences, which aligns with our understanding of interactions in the HRI domain. In TICK, an interaction is represented as a tuple $\langle o, a, e, d\rangle$, where *o* denotes the physical objects, including the agents (i.e., robots and humans), *a* represents the robot’s action and the human’s reactions, *e* indicates the event in which the interaction takes place, and *d* captures the attributes influencing the interaction, such as human personality. Formally, the representation of interaction in FOL is as follows:

$$ Interaction(p) \equiv dul.Quality(q) \wedge \exists o, a, e, d $$

$$ Physicalobject(o) \wedge hasParticipant(p, o) \wedge $$

$$ Event(e) \wedge hasParticipant(p, e) \wedge $$

$$ Action(a) \wedge dependsOn(p, a) \wedge $$

$$ Description(d) \wedge satisfies(p, d) \wedge $$

$$ \forall i (interaction(p) \wedge satisfies(s, n) \rightarrow hasPreferredInteractionStyle(pi, p)) $$

where *pi* refers to the preferred interaction style.

This definition states that an interaction (p) involves participants (o) that encompass both robots and humans. They engage in an event (e), and the best action for the robot in the subsequent interaction can be inferred based on their behaviour/actions (a), while satisfying specific description requirements (d). The preferred interaction style (pi) is associated with the interaction, allowing for the specification of the most suitable mode of interaction.

### Domain Ontology Level

We present a domain ontology specifically designed for the kitchen domain, focusing on concepts related to meal and drink preparation. The ontology, depicted in Fig. 4a, enables robots to anticipate and perform tasks by inferring human intentions during interactions. It encompasses various properties that describe objects within the kitchen domain and illustrates the interplay of physical objects, actions, and information realisation during task execution in this domain.

The physical objects in the ontology are categorised into Cutting tools, Measuring tools, and Preparation tools. More specifically, Cutting tools are characterised by the property of having a *sharp edge*, which is associated with the concept of Extreme Harmful. Measuring tools, on the other hand, possess the property of having breakable material, corresponding to the concept of Harmful. Finally, Preparation tools are classified as having unbreakable material, which aligns with the concept of Slightly Harmful. Figure 4b provides a visualisation of the Kitchen domain. Each category within the ontology has its own set of rules. For example, the Measuring tool class is defined as follows:

$$\begin{aligned}  &   MeasuringTool (mt) \equiv \\  &   \quad dul.PhysicalObject(o) \wedge \\  &   \quad hasBreakableMaterial(mt, m) \wedge \\  &   \quad \quad causes(mt, eh) \end{aligned}$$

where *eh* and *m* refer to the Harmful and Material concepts, respectively. This definition captures the essence of measuring tools by associating them with breakable material, leading to a harmful level of risk.

To illustrate the proposed scenario, when an object is detected as a Mug using the YOLO object detection system, it is assigned to the Mug class within the ontology and inherits the properties associated with that class. Consequently, the object is classified as a measuring tool with potentially breakable material, indicating a harmful level of risk. This classification methodology is employed to categorise all objects encountered in the kitchen domain.

**Fig. 5:**
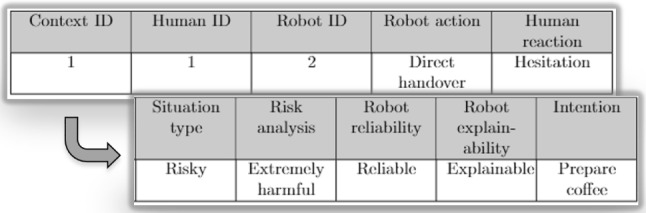
Context representation table. An example of episodic memory structure. It is structured to store data about the context of a situation. Each context has a unique ID and contains information about **a** which humans and robots are participating in the current situation, **b** which object is used for a given task, (c) what is the robot’s action, and **d** what is the human reaction. **d** what is the situation analysis, includes the situation type, the risk, intention, and reliability analysis

### Instantiation Knowledge: Interaction Memories

We exploit episodic memory as the version of interaction memory in TICK. Episodic memory is used to facilitate the agents to capture and store dynamic information regarding the environment and its entities, which can be retrieved when necessary. The Episodic memory system requires a constructive capacity to compile individual details into a coherent memory of a given episode. Explicitly, we structure the memory to store data about the context of a situation, with each context having a unique identifier and containing information about (a) participating humans and robots, (b) the object utilised for a particular task, (c) the action performed by the robot, and (d) the reaction exhibited by the human. Moreover, the reasoned information regarding the type of situation, risk level, robot reliability, robot explainability, and human intention. This information is employed to determine the optimal course of action for the robot based on the human’s prior experience.

As illustrated in Fig. 5, we designed a database table to store perceived data in a generic fashion using key-value pairs. Additionally, we created other database tables to store reasoned data associated with an episode. To establish a connection between a row in the database and other data, we require the instance of the episode, the time, and the collected data. This approach facilitates the bundling of data obtained simultaneously. Subsequently, this information is utilised to deduce trust as a binary variable, implying that trust will be considered true only if all components of trust align with the ontological definition (graph).

### Knowledge Reasoning Layer: Competency Questions

Our approach for monitoring human and robot interactions involves a reasoning mechanism that updates the robot’s understanding of the world, including the task and the situational assessment as well as human intention. To develop this reasoning procedure, a set of competency questions has been defined to be addressed, not in order and namely:

1. What constitutes human intention? This relies on sub-questions:
  What is the human’s belief about the robot?
  Which task has a goal of X?
2. What is the task being shared by the human and the robot?
3. Which action should be selected by the robot to achieve the human goal?
4. What is the situation type in which the human and the robot are involved?
5. Which object is involved in the task?
6. Is the robot or human vulnerable in this situation?
7. How can the robot explain a risky situation to a human?
8. How can the robot personalise its behaviour to accumulate human trust? (i.e., Which interaction style does the human prefer? )

These questions are proposed to enable human-centered reasoning that allows the robot to comprehend the human’s state, goal, belief, intention, and the preferred interaction style during the interaction. Additionally, robot-centered reasoning allows the robot to understand the shared task with the human, its responsibilities, including the actions to be performed, reliability and vulnerability, and the best behaviour to personalise its behaviour to accumulate human trust. Moreover, object-centered environment allows the robot to understand the state of the environmental entities. The combination of these factors makes the robot aware of the situation and how its actions can be performed. Examples of the implementation of these questions have been introduced in Sect. 4.4.1 to illustrate how they are grounded in a real scenario.

**Fig. 6:**
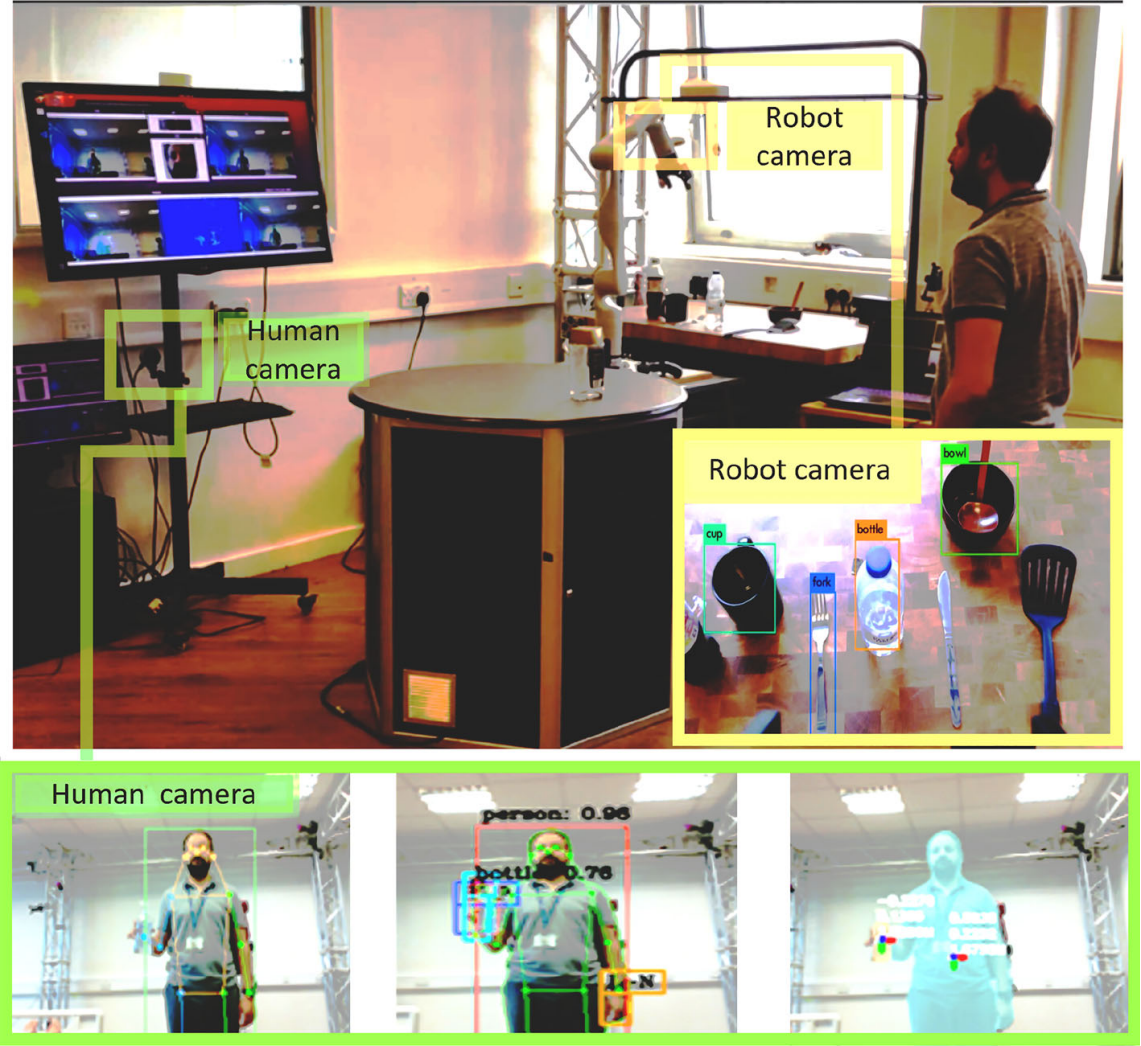
An example of a human and a robot interacting together in the kitchen scenario

## Case Study

To instantiate the TICK framework in a real HRI scenario, we consider using an object handover task, where the robot passes an object to a human, as an example task given its applicability in multiple scenarios, including home, healthcare (e.g. surgical), and manufacturing environments [44]. In this paper, the handover task is situated in a kitchen domain (two distinct scenarios). The objective of this case study is to showcase the functionality of TICK, particularly its ability to comprehend, assess trust, and personalise the robot’s behaviour towards the human participant.

We begin by providing an overview of our experimental setup, followed by a detailed explanation of how the TICK architecture is instantiated and the information flow within our scenario. Subsequently, we delve into the implementation of TICK’s reasoning capabilities and explore how trust influences the personalisation aspect of the system.

###  Use Case Overview

Let’s consider a human and a robot interacting with each other in a shared handover task of an object in a kitchen setting, as shown in Fig. 6. In particular, the perceived risk associated with the handover of a knife is likely to be higher especially when the human lacks prior experience with the robot’s execution of the task, compared to the handover of a cup which is perceived as less risky. It is hypothesised that the impact of this situation on the perceived risk influences the level of trust that humans place in the robot. Similarly, the impact of analysing the current situation will help the robot to perceive its vulnerability, reliability, and ability to infer human intention are also considered as the factors that influence human trust. Several steps are proposed to improve the robot’s ability to infer trust and its factors in such situations. Firstly, the robot needs to establish a common understanding and representation of the concept of trust with the human participant, including the factors that influence it. Secondly, the robot must acquire knowledge through the use of an intelligent perception system, which includes the perception of the environment and human states, resulting in context-aware robots. Thirdly, the robot should have a reasoning mechanism enhanced by experience-based interaction memories to remember previous interactions and ground information, which can be used to predict and personalise its behaviour in future interactions as well as recommend proper advice to the human in, for example, risky situations. A parameterised action, such as a slow-speed handover, is suggested as a means to enhance the human’s trust in the robot. Despite these advancements, the automatic evaluation and inference of the trust level of humans during the interaction with robots remain a challenge.

### Experimental Setup

The experiment has been done at the Personal Robotics Lab ,^4^ and it is composed of:

- The Kinova Gen3 arm is equipped with 2F-85 Robotiq grippers.
- Two tables, the preparation table which is used to prepare the task by the human, and another table, the Kitchen table, in which all objects are located in front of the robot.
- A set of kitchen objects including knives, bowls, plates, spoons, cups, bottles, forks, as well as fruits and coffee jars, etc.

Before describing the proposed scenarios, a set of assumptions have been taken into account.

- The human can not access the area of the kitchen table.
- Human applies either *pickUp* or *move* actions to the target object when starts the task to express their goal.
- The data extracted from the multi-modal perception system is responsible.

**Fig. 7:**
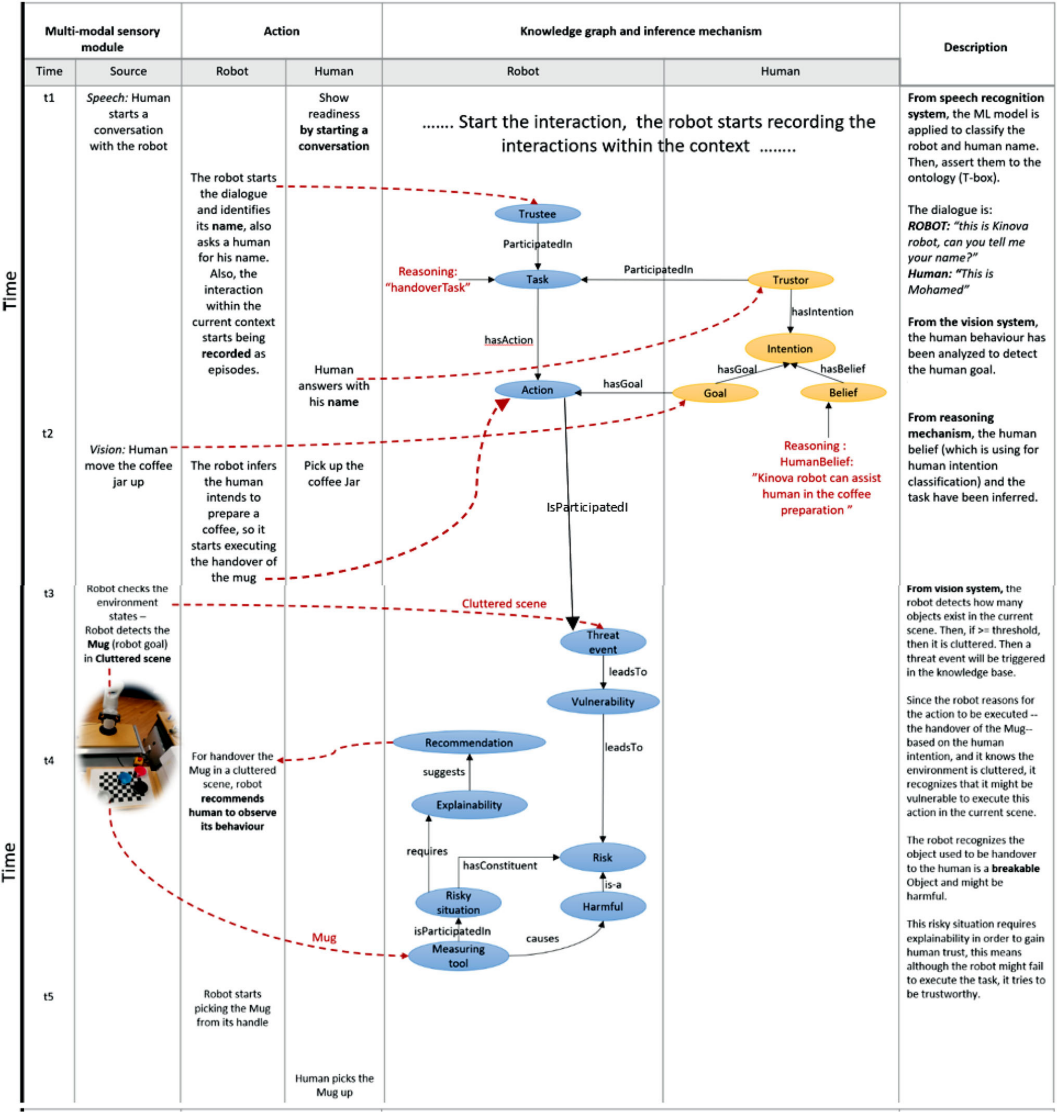
The flow of information between the knowledge acquisition’s modules to build understandable knowledge of the working domain including the current scene of the robot

### Implementation

#### Vision Module

In the TICK system, as shown in Fig. 6, we utilise the techniques: (a) a body pose estimation method to obtain a representation of the current human body pose of the user and use depth information to detect the distance to the robot, which can be used to monitor and understand user reactions to the robot’s actions; (b) a hand pose estimation to recognise contextual information, such as detecting whether an object has been picked up by the user; and (c) a scene understanding to describe the semantic features, such as the location of physical objects participating in the current situation. The (a) and (b) are used human recognition, while the (c) is used for object recognition. Explicitly, two cameras have been used for distinct purposes. The first camera is dedicated to recognising human (re)actions, while the second camera is mounted on the top of the robot arm and is exploited for detecting and localizing physical objects in the environment.

***Human Recognition***

To estimate the body pose and hand pose, we use the open-source library Message Passing Framework (MPF) [45]. This framework employs a body pose estimation method  [46] to obtain a representation of the current human body pose of the user and uses depth information to detect the distance to the robot, which we used to monitor and understand user reactions to the robot’s actions. For instance, when the user’s state is inferred as being near or far from the robot, the robot can determine whether the user hesitates or not. This state is currently analysed based on the pose estimation and proximity (depth) to the robot’s visual sensor.

To demonstrate the application of our proposed algorithms in robot-human handover scenarios, we initially tested the ResNet18 Convolutional Neural Networks model [47] using a limited number of videos. Since subjective human behavior recognition, such as hesitation recognition, is a relatively new area in computer vision research with no publicly available datasets, we recorded our own videos featuring hesitation and non-hesitation behaviors. These videos captured human reactions to a robot’s prior action, such as object handover.

To address overfitting concerns with small datasets, we employed two strategies: fine-tuning and encoding videos into images. With fine-tuning, we loaded the pre-trained parameters of the ResNet18 model and fine-tuned only the last three layers using the encoded hesitation video examples. The encoding process involved generating proximity graph images by measuring depth at the center point of the human region in each frame of the video. This approach enabled the deep learning model to focus specifically on behavioral features like proximity without needing to learn all the complexities of raw video data. Additionally, deep learning still had the capability to capture more features from the proximity information, such as speed and hesitance.

To establish a baseline for hesitation accuracy, we trained and tested the ResNet18 model using all video examples collected from two individuals and subsets specific to each individual. The results showed that the model’s performance in recognizing subjective behaviors degraded when trained on the entire dataset compared to training on individual-specific subsets.

The MPF is a collection of advanced techniques that can be used as the main sources in HRI scenarios. The framework consists of three state-of-the-art modules, namely, hand-object state prediction [48], human pose estimation [48], and video instance segmentation methods [49]. Each module outputs a different result, with the hand-object state recognition module predicting the bounding box of each hand and interacting objects with the hands, along with hand-contact states. The human pose estimation module predicts the positions of body joints and the corresponding predictive confidence scores for each joint. The video instance segmentation module predicts the instance region of an object and the ID of the instance, which confirms if the instance in the current frame is the same instance appearing in other frames in the video input. The main advantage of using the MPF is its stable output, with a low amount of noisy entries to the memory system, which is beneficial for HRI scenarios where the interaction is long-term and requires capturing context over time.

***Object detection:***

To estimate the semantic features, we utilise the rosified version of Yolo  [50], which is a real-time object detection deep learning open-source library. Object detection produces bounding boxes of objects, prediction scores of individual bounding box, and segmentation region within the bounding boxes. This library is trained using the COCO dataset  [51], which includes objects in the kitchen domain. The algorithm works by dividing the image into a grid and predicting the class and location of each object within each grid cell. By doing this, YOLO is able to perform object detection in real-time and with acceptable accuracy. To estimate semantic features, we first obtain the image using the 3D camera mounted on the robot. This image then be passed to the YOLO algorithm, which will identify objects within the image and their corresponding class labels. On top of that, we use the depth information obtained from the 3D camera to estimate the size and distance of the identified objects.

#### Speech Module

In TICK, we use mainly the speech system to (a) identify the humans and the robots participating in a joint task. (b) check the human readiness signal indicating the start of the interaction (this will help the robot to start recording its interactions with the participants). (c) explain, as an explainability method within the robot’s capability, the situations that might require clarifications for the human to avoid risks.

**Fig. 8:**
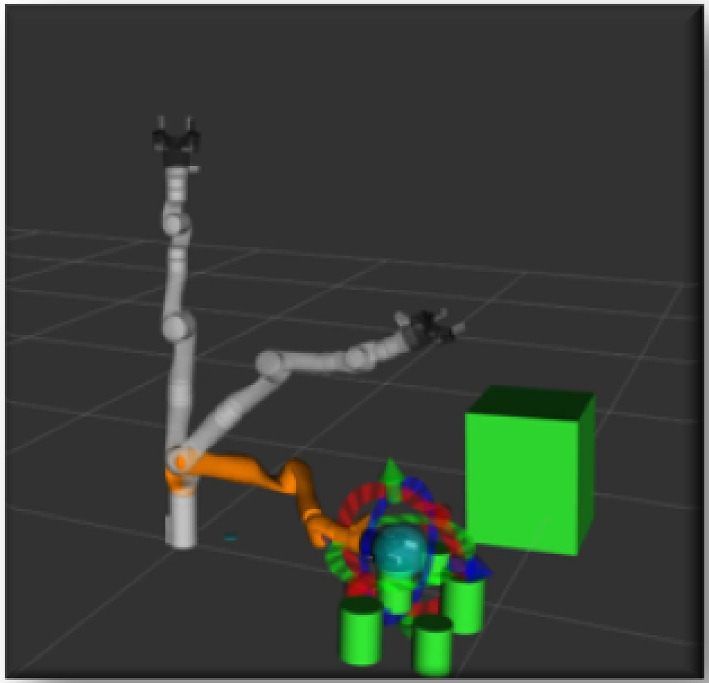
In Tick, a model-based approach is used to represent objects as CAD meshes and recognized as bounding boxes. Specifically, kitchen objects like cups and bottles are represented as CAD meshes and recognized as bounding boxes, which serve as models for the motion planner. These representations are used for reachability and collision checks. As an example, this is a planning scene of the coffee preparation task. The motion planner plans a feasible trajectory towards a grasping pose of the mug. If failure is reported, the robot updates its reliability in this context

The implemented algorithm for this purpose outlines a process for speech synthesis and recognition, with the goal of identifying a specific keyword and extracting the speaker’s ID from their speech. Speech Synthesis involves creating human-like text-to-speech output, while Speech Recognition refers to the process of interpreting and understanding human speech. The algorithm begins by obtaining the human speech through a microphone and then generating a response based on the context. It then uses the spaCy NLP library  [52] to analyse the speech, dividing it into sentences and returning all words to their infinitive root form. The algorithm then maps the extracted words to the keyword and checks if the keyword is present in the speech. If the keyword is detected, the signal is sent. The algorithm then classifies the speech and extracts the human ID from the classification output. For example, let’s say we want to identify the presence of the keyword “ready” in an utterance. First, we can tokenize the input text using the spaCy tokenizer. Then, we can iterate through each token in the sentence and check if it matches the keyword “ready”. If a match is detected, we can record the presence of the keyword and its position in the sentence. Additionally, we can use spaCy’s part-of-speech tagging to filter out words that are not relevant to the keyword. For instance, if we are only interested in the keyword “coffee” when it is used as a noun, we can check if the token’s part-of-speech tag is “NOUN” before recording the presence of the keyword. The process continues until the human–robot interaction is completed.

#### Geometric Planning Module

In TICK, as shown in Fig. 8, we utilise a planning module to allow their robot to plan its task at the motion level, taking into consideration the geometry of the objects present in a cluttered scene. To accomplish this, we use a motion planner, MoveIt [53], which provides services to reason on geometric information about the objects in the scene. MoveIt is an open-source motion planning tool that enables planning under geometric constraints. Our main goals of using geometric planning is to check the feasibility of the handover primitives and generate a feasible trajectory towards grasping a specific goal. For this purpose, we use the RRT-Connect motion planner to generate a path between two configurations in their experiment. Additionally, we use it for reporting the robot’s reliability, which means the robot fails to plan the primitive if it occurs.

The information acquired from the knowledge acquisition layer is collected and then asserted to the knowledge base to build the knowledge as discussed in the following section.

### How Does the System Work?

This subsection outlines the information workflow utilised to achieve designated tasks. The process commences with the human uttering the keyword “Ready” to the robot, as depicted in Fig. 7. The robot subsequently reciprocates by providing details about itself and soliciting similar information from the human. The extracted information from the conversation such as the humanID and the robotID are automatically mapped to the concepts Trustor and Trustee respectively. The tokens used in this exchange are classified. The information obtained is then leveraged to deliberate on previous experiences with a particular HumanID, such as interaction style, trust level, among other factors.

To initiate the task, the robot needs to infer the human’s sub-goal and belief, which requires a process of understanding the human’s intention. The human sub-goal is inferred using a vision algorithm [45] that can recognise the human action and the object the human is grasping. For instance, when a human picks up a coffee jar, the vision module can recognise the pick-up action performed by the human and then detect the object picked up. Once the task and human intention are identified, the robot utilises reasoning to infer which action should be applied and which object is required to be delivered to the human.

The identification of the situational state, such as clutter, is facilitated through the utilisation of a vision algorithm (YOLO). Building upon this algorithm, we develop a function that classifies the current state of the environment, such as determining whether it is cluttered or not, based on the presence and number of objects within an area of interest. Furthermore, the assessment of the robot’s reliability is accomplished by leveraging the geometric planning module. Prior to task execution, an environment model is constructed, and a motion planning service incorporating collision detection is employed to generate a trajectory towards a specific goal. If the planning phase successfully generates a trajectory, a signal is triggered to activate the reliability mode of the robot for executing the task in the current situation.

In order to effectively operate within a particular environment, such as a cluttered setting, the robot must engage in a process of reasoning to evaluate its susceptibility to potential damage and assess the vulnerability and risk associated with transferring objects to humans under specific circumstances. To achieve accurate vulnerability assessment, we analyse the robot’s historical performance when executing comparable tasks in similar tasks. Furthermore, the classification of risk levels pertaining to the environmental entities involved in the current situation is depicted in Fig. 4b. The degree of risk is categorised into three distinct levels: slightly harmful, harmful, and extremely harmful, each of which is assigned an appropriate risk level designation of low, medium, and high, respectively.

The subsequent subsection presents a comprehensive exposition of the detailed implementation of all the aforementioned reasoning queries.

#### Heterogeneous Reasoning Mechanism

A series of general queries (competency questions presented in Sect. 3.7) have been applied to the knowledge representation layer to derive a set of fundamental questions for trustworthy HRI. These queries are categorised into two types: symbolic and non-symbolic. Symbolic queries are aimed at querying the ontology to understand the trust-related factors including human goals, human beliefs, robot vulnerabilities in specific situations, situation classification, and proposing recommendations based on robot capabilities. The output of symbolic queries is then stored in the database as dynamic knowledge that represents the status of the world in a specific event, which later becomes a source for non-symbolic queries. Non-symbolic queries are used to retrieve information on past interactions, such as preferred interaction styles, from the database. Some examples of both query categories will be presented as follow.

***Symbolic queries***

The following symbolic description logic (DL) query aims to retrieve the task that has a specific goal, which is defined as, for instance, the human picking up the coffee jar. The query is executed on the ontology, which stores information about the domain in a structured format. The query contains three conditions: (a) the task has a human and a robot participant, (b) the task has a specific goal of the human picking up the coffee jar, and (c) the query result is the instance of the task that satisfies the above conditions.

***DL query***

Which is the task that has a goal e.g., *“human picks up the coffee jar”*?

$$\begin{aligned}  &   (hasParticipant \, \, {\textbf {value}} \, \, human) \,\, {\textbf {and}} \\  &   \quad (hasParticipant \, \, {\textbf {value}} \, \, robot) \, \, {\textbf {and}} \\  &   \quad (hasGoal \, \, {\textbf {value}} \, \, human-picksUp-coffee-jar) \end{aligned}$$

**Query result:**

      **Instance:** $$coffee-preparation$$

The following DL query aims to retrieve the belief of a human participant who was involved in a specific task, such as the coffee preparation task and had a goal, such as picking up the coffee jar. The query output is a data property that represents the human belief regarding the robot’s capability to assist in the coffee preparation task. This data property is presented in natural language and reflects the human’s positive perception of the robot’s ability to provide assistance in the task.

***DL query***

What is the human belief about the robot (e.g., Kinova) sharing the task (e.g., coffee preparation)?

$$\begin{aligned}  &   isBeliefOf \, \, {\textbf {some}} \,\, (Human \,\, {\textbf {and}} \, \, isParticipantedIn \, \, \\  &   \quad {\textbf {value}} \, \, CoffeePreparation) \, \, {\textbf {and}}\, \, \\  &   \quad (hasGoal \, \, {\textbf {value}} \, \, human Picks Up The CoffeeJar) \end{aligned}$$

**Query result:**

      **Data property:** *Can assist me in the coffee preparation task*

The following query aims to determine the human intention related to a specific task (e.g., coffee preparation). It asserts that the human agent’s participation in picking up a coffee jar is a prerequisite for achieving a goal related to coffee preparation. Additionally, the query states that the human agent believes that this action can assist in achieving this goal. The query result is a specific instance of a human intention: the desire to drink coffee.

***DL query***

What is the human intention?

$$\begin{aligned}  &   dependsOn(Agent\, \, {\textbf {and}} \, \, isParticipatedIn \, \, \\  &   \quad {\textbf {value}} \, \, human-picksUp-coffee-jar) \, \, {\textbf {and}} (hasBelief \, \, \\  &   \quad {\textbf {value}} \, \, Can Assist Me In The Coffee Preparation Task) \end{aligned}$$

**Query result:**

      **Instance:** $$human-wants-to-drink-coffee$$

The following query aims to determine the explainable capability of the specific robot (e.g., Kinova). The query asserts that there exists a method used by the robot, which is an agent that participates in the Coffee Preparation task. The query result specifies that the explainable capability of the Kinova robot is related to speech, which suggests that the robot is capable of providing verbal explanations or feedback regarding its actions or the task at hand.

***DL query***

What is the explainable capability of the robot (e.g., Kinova)?

$$\begin{aligned}  &   isTheMothodOf \, \, {\textbf {some}} \, \, (Agent \, \, {\textbf {and}} \\  &   \quad \, \, participatesIn \, \, {\textbf {value}} \,\, CoffeePreparation) \end{aligned}$$

**Query result:**

      **Instance:** *Speech*

The query aims to determine whether the robot is vulnerable in a specific situation (e.g., a cluttered environment). It asserts that the ability of the robot to manipulate and navigate in a cluttered environment depends on the human agent picking up a coffee jar. The query also specifies that the robot’s ability to manipulate and navigate in a cluttered environment is constrained by the inability to navigate in cluttered environments, as realised by a specific capability or component called “canNotManipulateInCluttered.” The query result specifies whether the robot is vulnerable in the current situation or not, which means that the robot is at risk of being damaged or malfunctioning in a cluttered environment.

***DL query***

Is the robot vulnerable in the current situation (e.g., cluttered environment)?

$$\begin{aligned}  &   dependsOn \, \, {\textbf {some}} \, \, (Agent\, \, {\textbf {and}} \, \, isParticipatedIn \, \, \\  &   \quad {\textbf {value}} \, \, human-picksUp-coffee-jar) \, \, {\textbf {and}} \\  &   \quad \, \,(isRealizedBy \, \, {\textbf {value}} \, \, canNotNavigateInCluttered \, \, \\  &   \quad {\textbf {and}} \, \, hasConstraint \, \, {\textbf {value}} \, \, clutteredEnvirtonment) \end{aligned}$$

**Query result:**

      **Instance:** *vulnerable*

The following query seeks to find the categorisation of the current situation based on the level of risk. Specifically, it looks for situations where a cutting tool is involved and creates extremely harmful effects, and an agent is present that has a vulnerability and is part of a threat event. The query can be paraphrased as “What is the categorisation of the current situation based on the presence of a cutting tool that creates extremely harmful effects, and an agent with a vulnerability that is part of a threat event?” The query result indicates that the current situation is classified as a “high-risky-situation”, which implies that it poses a significant threat to the safety of the agent and possibly other entities involved in the situation.

***DL query***

What is the categorisation of the current situation? (if risky, which level of risk the current situation is?)

$$\begin{aligned}  &   (hasSource \, \, {\textbf {some}} \, \, Handover cutting tool) \,\, {\textbf {and}} \,\, \\  &   \quad (hasParticipant \, \, {\textbf {some}} \, \, (Cutting tool \, \, {\textbf {and}} \, \, \\  &   \quad ( creates \, \, {\textbf {some}} \, \, Extremely harmful))) \, \, {\textbf {and}} \, \, \\  &   \quad (hasParticipant \, \, {\textbf {some}} \, \, (Agent \, \, {\textbf {and}} \, \, \\  &   \quad (hasVulnerability \, \, {\textbf {value}} \, \, vulnerability) \, \, {\textbf {and}} \, \, \\  &   \quad (isPartOf \, \, {\textbf {value}} \, \, threatEvent)) \end{aligned}$$

**Query result:**

      **Instance:** $$high-risky-situation$$

The following query aims to provide a proper recommendation for a given situation based on certain conditions. The query is checking whether the situation is of the type “riskySituation” and whether it has a “highRiskLevel” by using the DL axioms of “hasSituationType” and “hasPart”. The query is looking for instances that satisfy these conditions. The query result indicates that the recommended action in this situation is to “Observe My Behaviour and Keep Proper Distance”. This recommendation is based on the high-risk level of the given situation and suggests that the agent should closely monitor the behaviour of the surrounding entities and maintain a safe distance to avoid any potential harm.

***DL query***

What is the recommendation? (i.e., in the current situation)

$$\begin{aligned}  &   \text {hasSituationType} \, {\textbf {some}} \, ( \text {riskySituation} \, {\textbf {and}} \, \\  &   \quad \text {hasPart} \, {\textbf {value}} \, \text {highRiskLevel} ) \end{aligned}$$

**Query result:**

      **Instance:** *ObserveMyBehaviour*

      **Instance:** *KeepProperDistance*

The aforementioned queries can be leveraged and reused across various HRI tasks, providing a versatile and adaptable approach for extracting relevant information and insights.

***Non-symbolic queries***

The symbolic queries are generally used in the TICK system to infer information either at the metaontology level such as the meaning of concepts like Trust and its factors (making use of the definitions of these concepts in the knowledge representation layer) or at the domain level such as information regarding specific tasks such as coffee preparation. Meanwhile, the non-symbolic queries are used to infer information about the instantiation ontology level such as human preferences in a specific context.

Some queries are presented as general examples. The following query retrieves all information in the $\textit{contextID}$ row from the $\textit{contextTable}$ in a database where the $\textit{humanID}$ value matches a specific variable (presumably input automatically by the multi-modal sensor) and then returns only the first matching result. The option to select specific content(s) is also available following the same strategy.
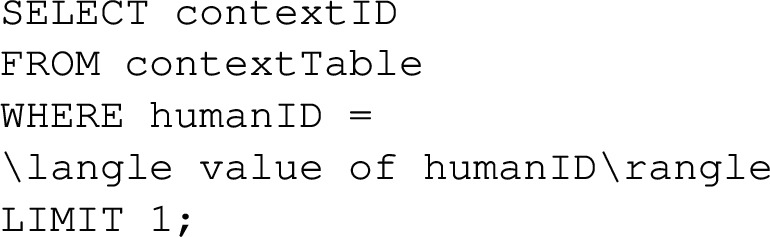


The following query is used to add a new record to the $\textit{contextTable}$, with the specified values for each column. The data type of each value must match the data type of the corresponding column in the table. These Values are automatically asserted from the multi-modal sensor module.
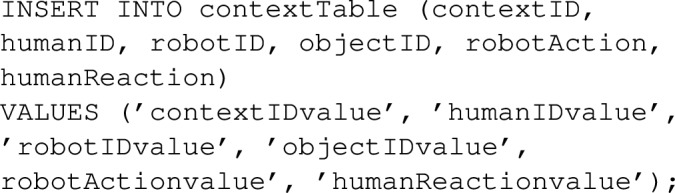
 Similarly, as shown in Fig. 5, reasoned information such as situation type, risk analysis, etc. are also asserted automatically to the database using the symbolic reasoning queries mentioned above.

**Algorithm 1:**
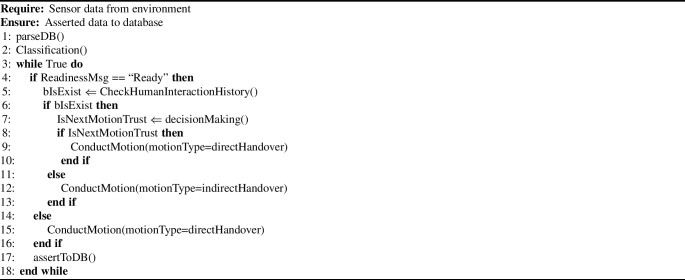
Manager Algorithm

### The Manager Algorithm

The TICK system possesses a broad applicability across various tasks in the field of HRI rather than being tailored to a specific task. Its versatility allows it to accommodate multiple tasks, necessitating some adaptations related to the environment’s description through domain ontology. The aforementioned modules, including perception, planning, and reasoning, have been encapsulated to provide services that are utilised by the manager algorithm 1. The manager assumes the responsibility of invoking these services to autonomously execute shared tasks. The manager algorithm operates by continuously acquiring sensory input from the surrounding environment, engaging with ontologies to comprehend concepts and interaction memories, and subsequently personalising the robot’s behaviour to suit a specific user if trust is established.

Line 1—encompasses the subroutines responsible for parsing the two distinct databases, the ontology and the interaction memories. The services have been implemented to facilitate the loading of ontological models, as explicated in (Sect. 3.4—symbolic query), thereby enabling the robot to comprehend and infer information pertaining to the meaning of concepts. Furthermore, the services encompass the provision of a comprehensive semantic depiction of the kitchen environment. Additionally, other implemented services encompass the ability to query the interaction memories, as elucidated in (Sect. 4.4.1 non-symbolic queries), thereby furnishing insights into past interactions and facilitating the updating of information within this memory.

Line 2—This segment encompasses the classification subroutines for both vision and speech processing. In vision processing, the traditional YOLO (You Only Look Once) network is utilized for object classification. This method involves segmenting an input image into a grid of cells. Each cell then predicts a predefined number of bounding boxes that might contain objects. Subsequently, the YOLO algorithm assesses the likelihood of each bounding box containing a specific object class (e.g., person, knife, cup) and the confidence level in the accuracy of the predicted bounding box.

As for speech processing, it integrates both Speech Synthesis and Speech Recognition learning techniques. The speech algorithm is engineered to recognize and interpret human speech, categorizing it into various classes and extracting pertinent information, such as the human’s ID. The tokens from both participants (i.e., human and robot) are subsequently categorized and mapped to the ontology as Trustor (HumanID) and Trustee (RobotID).

Within lines 3-18 of the algorithm, the following operations are performed: upon reception of a keyword signal, such as a “Ready” message, the robot proceeds to examine its interaction memory to determine if any prior engagements with the human have occurred. If records of previous interactions are identified, the algorithm engages in a decision-making process to select an appropriate motion strategy based on the human’s demonstrated level of trust. In scenarios where no interaction history exists, the algorithm executes a direct motion, incorporating geometric planning, while evaluating the human response as an indicator of trustworthiness. The motion services encompass reasoning about object geometry, where the initial and goal states are provided to the robot, and the planner generates a feasible trajectory towards the goal. Subsequent to each interaction, the resulting data is stored in the database, allowing the robot to access previous reactions and adapt its behaviour accordingly for subsequent interactions with the same individual. This adaptive behaviour allows the robot to optimise its interaction strategies based on past experiences and facilitate improved engagement with the human user.

**Fig. 9:**
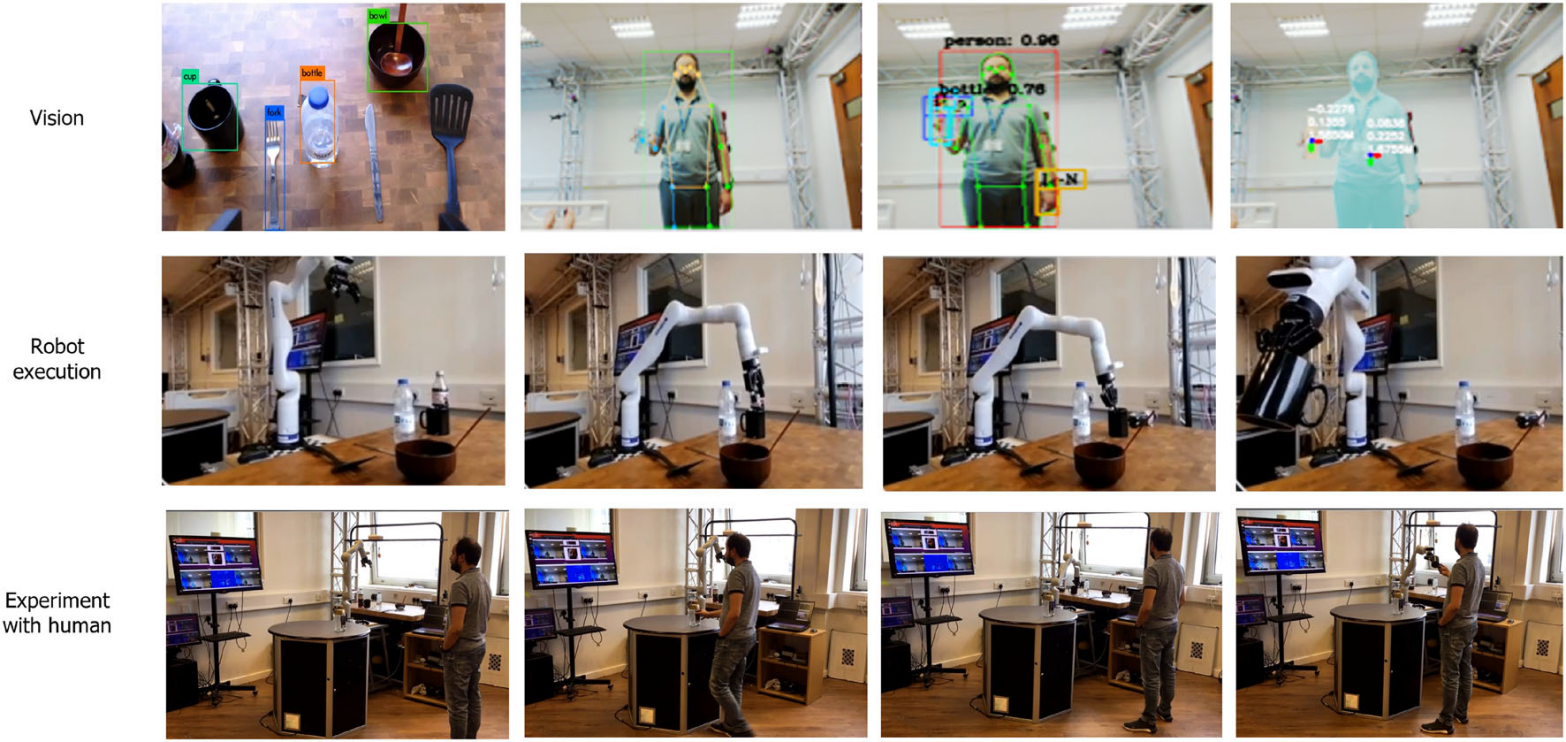
The three different scenes of the experiment. The first row shows the extracted features from the perception module. The top left is the image from the camera attached to the robot arm (robot’s vision) to detect the objects’ states. The rest of the row shows the features extracted from another camera fixed in the environment to recognise the humans’ behaviour such as body pose estimation, hand pose, and proximity. The second row shows how the robot handover the *cup* to a human in the execution phase. The third row shows the sequence of the whole experiment executed with the human. The video link of the experiment is shorturl.at/dswZ2 and shorturl.at/ikzLV

### Trust Estimation

Trust is a complex concept that encompasses multiple dimensions and can be perceived and evaluated using a comprehensive formula. Trust evaluation in TICK is formalised as process as it involves several distinct steps that should be systematically followed to arrive at a comprehensive understanding of trust. Firstly, we identify the key features that impact trust factors, such as proximity and object type. Next, these features are normalized and linked to their respective definitions, like “Risk.” Finally, trust itself is determined as the satisfaction with all factors within the trust definition. Each of these steps requires careful consideration, mathematical computations, and logical reasoning to accurately evaluate and quantify trust. This systematic approach ensures that the evaluation is thorough, methodical, and based on sound principles, highlighting the process-oriented nature of trust assessment.

Drawing inspiration from previous work [54], trust (*T*) is quantified as the product of *h* and *ptf*, denoting the robot’s experience about its performance or about its interaction with a specific user and the perceived trust factor, respectively:

1 $$\begin{aligned} T = \textit{h} \times ptf \end{aligned}$$

The value of *h* can be estimated, as shown in equation  2, by considering various factors, including the robot’s self-assessment, behavioural measures, and performance indicators [4] (as discussed in Sect. 1). *Self-assessment* refers to the robot’s evaluation of its own trustworthiness in relation to its components such as its perception or manipulation capabilities (as discussed in Sect. 5). The *behavioural measure* is assessed by examining the level of engagement, which, in our scenario, is gauged through proximity and adherence to the robot’s recommendations in high-risk situations. Proximity captures human behaviour, differentiating between “hesitation” when the human is near the robot and “no hesitation” when the human keeps a distance. Furthermore, the interpretation of human reactions can be calibrated in cases where the human follows the robot’s recommendation “keep a proper distance” despite increasing distance from the robot, transitioning from “hesitation” to “no hesitation”.

2 $$\begin{aligned} h = \sum _{i=1}^{n} ux_i + f(sa, bm, pi) \end{aligned}$$

Where:

- $ux_i$: User experience component $i$ with the robot.
- $n$: Total number of user experience components.
- $f$: Function representing the estimation process for trustworthiness, considering self-assessment (*sa*), behavioural measures (*bm*), and performance indicators (*pi*).

The *performance measure* evaluates the number of errors made, serving as an indicator of the success rate. This measure is estimated based on human confirmation at the end of the experiment, such as the human expressing satisfaction with the robot’s personalised behaviour.

The other term *ptf*, as shown in equation 3, encompasses trust-related aspects such as Situation analysis, Reliability, Vulnerability, Risk analysis, Intention and Explainability. The factors have been estimated through the heterogeneous inference mechanism presented in Sect. 4.4.1 and stored later in the DB. The user’s perception of the available technical information significantly contributes to their trust in the system.

3 $$\begin{aligned} \textit{ptf} = f(\textit{ux}_{\textit{r}}, \textit{fu}, \textit{rf}, \textit{os}) \end{aligned}$$

where:

- $\textit{fu}$: Functionality of the robot, reflecting its ability to perform tasks effectively and efficiently.
- $\textit{rf}$: Robot reliability, indicating the consistency of the robot’s performance.
- $\textit{os}$: Overall Satisfaction with the robot, capturing the user’s overall impression.

Each component affecting the *T* in the formula takes a value between [0, 1]. Then normalise it.Normalisation of data was performed using the following formula:

$$\begin{aligned} \text {Normalised value} = \frac{{x - \mu }}{{\sigma }} \end{aligned}$$

where: $x$ represents the data value, $\mu$ denotes the mean of the dataset, and $\sigma$ represents the standard deviation of the dataset.

When analysing normalised data, certain patterns emerge. A normalised value greater than 0 suggests that the corresponding data point exceeds the mean. Conversely, a normalised value less than 0 indicates that the data point is below the mean. In particular, the normalised value provides insight into the number of standard deviations by which the original data point differs from the mean. Each normalised value within the retrieved data from the database aids in assessing the proximity of a specific data value to the mean. A small normalised value signifies proximity to the mean, which interprets as “Trust”, whereas a large normalised value indicates significant deviation from the mean, which interprets as “No trust”. The “Trust” and “No trust” labels are then stored in the DB for subsequent future interactions.

### Scenario One: Coffee Preparation

Figure 9 presents a series of sequential snapshots illustrating the coffee preparation task, wherein the robot interacts with a human by handing over a cup based on the human’s intention. The interaction commences with the robot utilising a speech recognition system 5 to classify human tokens and speech, following a pre-designed dialogue that commences with the keyword “ready,” which is recognised by the robot. Subsequently, the robot introduces itself to the human and requests information such as the human’s name to cross-reference the stored past knowledge in the database (represented by the context representation table in Fig. 5) to infer previous interactions with the individual.

**Fig. 10:**
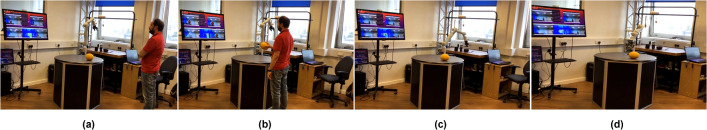
The sequence of the cutting fruit scenario. **a** shows the setup of the experiment. The table in front of the robot includes different kitchen objects such as a knife, cup, fork, etc. Meanwhile, the other table, in front of the human contains fruit. **b** The human starts moving the fruit to describe his intention to the robot. **c** The robot infers the type of object required for cutting the fruit and starts picking it up. **d** The robot executes the indirect handover to the human by putting the knife on the table. For safety, the robot slows its speed and recommends that human keep a proper distance. The video link of the experiment is shorturl.at/dknUY

Throughout the task, the robot monitors the human’s hand gestures, including the object held by the human, utilising a message-passing framework [45] to discern the human’s intention and select the appropriate object for the task. The robot employs an open-source library [50] to classify the objects on the table situated in front of it, whereby the library extracts the 2D location of the objects. In our implementation, we integrate an additional library 6 to facilitate the extraction of the 3D location. All object and camera transformations are computed relative to the world frame. The robot engages in symbolic reasoning to determine the necessary actions for the task and employs the MoveIt [53] framework to generate collision-free trajectories for each action, leading to the object’s grasping pose.

To ensure the robot remains aware of the current situation, various reasoning queries are employed, including risk assessment, robot vulnerability, and reliability, which dictate whether recommendations should be provided to humans. Knowledge representation is accomplished using the Ontology Web Language (OWL) within the Protege ontology editor. 7 Individuals are asserted based on information derived from low-level sensory data. Prolog predicates are utilised to perform queries over the ontological knowledge. The SWI-Prolog system, along with its Semantic Web library, is employed to load and access ontologies represented in OWL using Prolog predicates. Additionally, a Python-based ROS (Robot Operating System) 8 interface is implemented to facilitate the query-answer process, establishing client-service communication.

### Scenario Two: Cutting Fruits

To demonstrate the adaptability of the system to diverse tasks, let us consider a scenario where the objective is to cut a fruit, as shown in Fig. 10, rather than engaging in coffee preparation. In this context, the robot is required to comprehend the distinctions between these tasks through the process of inference. Specifically, it needs to infer: (a) the human intention, discerning that the intended action is to cut a specific fruit, such as an apple; (b) the type of action to be executed, involving the handover of a knife (as opposed to handing over a mug in the coffee preparation scenario), enabling the human to perform the cutting task. Leveraging the inference capabilities of the TICK framework, the robot becomes aware of the appropriate action to undertake based on the identified task requirements. Moreover, the personalised action of the robot, the handover of the knife, towards a particular user, is influenced by the risk level associated with this action. As handing over a knife can pose a significant risk to the human, the robot needs to consider motion parameters, such as its speed, as well as provide recommending advice, such as maintaining a suitable distance. By incorporating these considerations into its behaviour, the robot aims to personalise its actions and cultivate trust with the human user.

The robot evaluates the environmental state, which, in this case, is characterised by clutter. It assesses its own vulnerability, reliability, and the associated risk. Subsequently, the robot offers recommendations to the human, suggesting actions to be taken in order to mitigate potential risks, such as observing the robot’s behaviour and maintaining an appropriate distance. The vision system extracts proximity features (as depicted in Fig. 9) to determine whether the human adheres to the robot’s advice. The information stored in the database is then updated or calibrated accordingly. For instance, if the human complies with the robot’s recommendations, it indicates a level of trust in the robot, which is recorded in the database.

**Table 1:** Case study: a human is sharing a handover task with the Kinova robot

Context ID	Robot action	Human action or reaction	Description
1	Robot recognises the Human’s token and asks for their name and asks to start the task	Answers “My name is X” and starts picking up the target object	After the robot analyses the situation (i.e., environment type, vulnerability, risk) and recommend something to the human, new interaction DB entry created with Human ID
2	PickUp the target object on the preparation table (default: optimal-direct unless the situation is an extremely harmful level of risk)	Human first listens to the robot’s recommendation, then receives the target object. For the coffee preparation scenario, the recommendation is *observe my behaviour* and he doesn’t show hesitation. For the cutting fruit scenario, the recommendation is *keep a proper distance* and he does follow the recommendation	Interaction DB entry updated: For the coffee scenario, Human X trusts the robot with direct handovers. For the cutting fruit scenario, Human X trusts the robot with indirect handovers
3	PickUp the target object and handover to the human	Human receives the object without hesitation	Trust level not updated; human X still trusts the robot

There is a set of kitchen objects used in the proposed scenarios, and the robot applies reasoning queries to identify which object is required for a specific task. The human depicts their intention of the object handover and the Kinova robot adapts its behaviour, based on the previous experience of how the robot interacts with this human, and how the human reacted to the robot’s action. The robot also considers analysing the current situation in this evaluation which results in recommendations for the current situation

### Example of the Personalisation of Behaviour Generation: Remembering Capabilities

Table  1 presents an experimental scenario involving the object handover task. The authors assign the handover sub-task to the Kinova robot, which is capable of adapting its behaviour based on previous experiences, including (a) how the robot interacts with a specific human and (b) how the human reacts to the robot’s actions. In this particular scenario, two approaches, namely direct and indirect handover sub-tasks, have been implemented at the robot motion level, both performed through a table.

In the depicted scenario examples, the robot system recognises that the direct handover method, which is considered optimal, has varying effects on the same human in different contexts, thereby influencing the level of trust exhibited by the human. For instance, in the coffee preparation scenario, despite the handover of a cup being a potentially risky situation, the human demonstrates no hesitation and follows the robot’s recommendation to “Observe my behaviour” (as indicated in Table 1—in Context ID 1 and 2). Conversely, in the cutting fruits scenario, the robot hands over an extremely risky object and advises the human to “keep a proper distance.” In this case, the robot assesses the level of trust based on whether the human adheres to its recommendation or not. Therefore, even though the human physically distances themselves from the robot, they still comply with its instructions, indicating a high level of trust.

Subsequent to these interactions (in Context ID 3), the robot updates its episodic memory to record the outcomes. In future interactions, these recorded outcomes inform the behaviour generation process, enabling the robot to generate personalised behaviour, such as selecting between a direct or indirect handover. It is important to note that the adoption of the indirect handover approach (employed in the knife scenario due to the elevated risk level) is not optimal, but it reflects a potentially lower level of trust exhibited by the user towards the robot. Detecting a preference for sub-optimal behaviours instead of safe and optimal behaviours can trigger an explanation stage, where the robot provides verbal assurances, aimed at increasing the human’s trust. This calibration process is employed to attain an appropriate level of trustworthiness in the robot’s action execution system.

## Experimental Results

Our evaluation encompasses three points:

1. The system’s performance, is specifically measured by the success rate of handover interactions between the robot and the human, considering the human’s preferences. This evaluation involves investigating the robot’s memory capabilities and the robot’s ability to recall previous interactions with the specific human.
2. The temporal efficiency of the robot’s task execution during interactions with the human. This assessment includes evaluating specific components to identify means of enhancing task completion speed. Moreover, evaluating trust which affects the decision of the robot’s personalisation.
3. The robot’s self-estimation which engages in performance estimation both prior to and during interactions, subsequently utilising this information to provide users with an overview of its historical performance. This assessment encompasses various aspects, such as perception, speech, motion, and grasping self-estimation.

To facilitate this evaluation, we compare the system against various baselines, each representing different scenarios wherein interaction memories and ontologies are incorporated in diverse scene contexts with varying object types. The primary objective of this evaluation is to acquire valuable insights regarding the overall effectiveness of the system and its potential to deliver tailored and trustworthy interactions. By thoroughly examining these aspects, we strive to gain a comprehensive understanding of how well the system adapts to individual human preferences, enhances efficiency, and fosters reliable and personalised human–robot interactions.

**Table 2:** Compare the performance of applying the reasoning capabilities of the TICK framework in the handover task

Scenario	World type	Parameter	Execution phase			
			Baseline1	Baseline2	Baseline3	Baseline4
Coffee preparation	No cluttered	Success rate%	10%	33%	88%	93%
		Avg. time (s)	30	28.5	197.4	71
	Cluttered scene	Success rate%	10%	50%	96%	90%
		Avg. time (s)	43	39.5	210.6	84.3
Cutting fruit	No cluttered	Success rate%	9%	19%	95%	98%
		Avg. time (s)	11.5	39.5	150.2	50
	Cluttered scene	Success rate%	3%	15%	89%	96%
		Avg. time (s)	36	39.5	259.2	68.6

We test this performance in the execution phase of the proposed scenarios

The evaluation encompasses two distinct environmental conditions, a cluttered and a non-cluttered environment in the two proposed scenarios. Throughout the evaluation process, the overall performance of the robot in the handover task was assessed by comparing it against four baseline scenarios:

- *Baseline 1*: The database modules (ontology and interaction memories) are not activated (no knowledge).
- *Baseline 2*: The interaction memory is activated, while the ontology is deactivated.
- *Baseline 3*: The ontology is activated, while the interaction memory is deactivated. In this case, we utilise the capability of ontology to store information about the participants instead of the interaction memory.
- *Baseline 4*: The databases (ontology and interaction memory) are activated. These baselines were analysed and their results are presented in Table  2. The purpose of evaluating the system with and without different database sources was to understand the impact of integrating these modules together in the long term. Additionally, the evaluation aimed to assess the effectiveness of the comprehensive framework and identify areas for improvement.

**Fig. 11:**
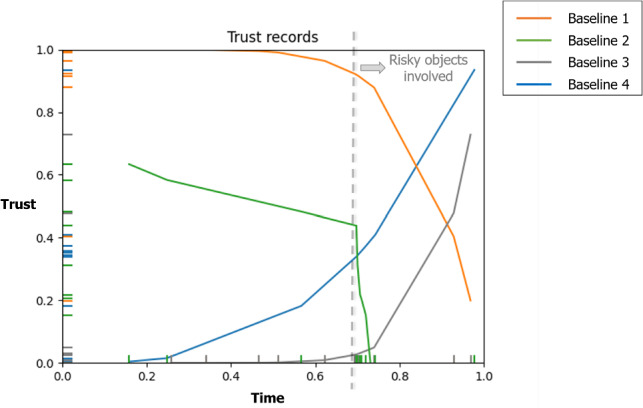
The estimation of the performance of the system of the four baselines

To ensure an unbiased assessment of the proposed system’s capability to infer trust in various scenarios and contexts, the system underwent a complete reset to a pristine state at the start of each experimental trial, devoid of any prior knowledge or understanding of trust specific to the user. This approach facilitated an impartial evaluation of the effectiveness of each individual module, as well as their adaptive and knowledge acquisition abilities during interactions. In each baseline, the system started afresh, relying solely on the information and feedback gathered throughout the ongoing interactions.

In order to gather sufficient data for analysis, a substantial number of interactions were conducted across the four baselines. We conducted 120 experimental trials, providing a diverse and extensive dataset to evaluate the performance of the system. These trials encompassed a wide range of scenarios and contexts, enabling a comprehensive assessment of the system’s ability to infer trust across different situations and, ensuring a diverse set of interactions to capture a broad spectrum of trust-related behaviours.

The findings of our analysis reveal that the lowest success rate 9 is observed when the robot lacks any form of understanding regarding trust and possesses no prior knowledge about the user. Subsequently, a marginal improvement is observed in the second baseline when trust measurement relies solely on proximity, disregarding other factors encompassed within the ontological model. This indicates that the robot’s comprehension of trust is contingent upon a single, albeit limited, factor, suggesting that if the robot is in close proximity to the user, it interprets this behaviour as preferable and endeavours to replicate it in future interactions. Although noticeable enhancements are observed in the preparation of the coffee task, it is noteworthy that the success rate diminishes in the cutting fruit task compared to the primary task. This implies that the robot fails to comprehend other significant factors such as risk and vulnerability, thereby impeding the accurate prediction of human trust. In the third baseline, we employ the ontological model equipped with its capacity to store and update user information. We observed an increase in the success rate across both scenarios; however, the execution time becomes impractical for online experiments. The reason behind this, as elucidated in the subsequent section, is associated with the performance of the reasoning mechanism. It’s noteworthy that in certain cases, working within a cluttered scene led to improved recognition of object names/IDs compared to scenarios involving a single object. This phenomenon was particularly evident in the coffee preparation task. We attribute the higher success rate in the cluttered scene variation to the intelligent perception module. The experiments were primarily conducted during daytime under varying lighting conditions, such as sunny or foggy weather. These conditions significantly affect object recognition, especially when dealing with objects of lighter colors. As depicted in Fig. 10, where the object appears yellow, fluctuations in lighting can introduce inconsistencies in object recognition, thereby impacting task performance. Our efforts to enhance the performance of the reasoning mechanism yielded positive results; however, we encountered the challenge of defining numerous specific relations to address uncertainties in reasoned information. Consequently, we posited that integrating both databases in the fourth baseline would yield superior outcomes in terms of success rate and time performance.

To evaluate the long-term effectiveness of the proposed baseline, we conducted an analysis of data collected over multiple days. Figure 11 presents the findings of this analysis. The results indicate that when the task involves the presence of risky objects, the trust level of the participants in baseline 4 tends to increase. This is attributed to the robot’s capability to recognise risky situations and subsequently provide relevant recommendations to address the inferred sense of risk.

**Table 3:** Comparison between the reasoners’ performance in terms of time (s)

Comparison of the reasoner performance	
Reasoner name	Reasoning time (s)
HermiT	511.8
Pellet	6.3
Konclude	1.2

As depicted in Table  3, the execution time of the implemented predicates of TICK has been assessed across different reasoning engines, HermiT  [55], Pellet  [56], and Konclude  [57]. Notably, the Konclude reasoner demonstrates the most efficient performance, with an average execution time of approximately 1 s per predicate. However, it is worth highlighting the unexpected observation that the HermiT reasoner exhibits significantly longer execution times, taking approximately 8 min (further elaborated in the comments in Sect. 6). Consequently, the evaluation of TICK performance, presented in Table  2, leverages the superior performance of the Konclude reasoner.

On the other hand, we also focus on the automation perspective while evaluating trust, for this purpose, TICK framework characterises trustworthiness as the collective performance of the robot and its individual components, necessitating the participants’ confidence in the robot’s capabilities during the interaction. This helps the robot to explain to the users its proficiency. To establish the robot’s knowledge-based proficiency regarding the performance of its components, a two-fold approach consisting of a preliminary stage and an interaction stage has been devised. The preliminary stage enables the robot to develop an estimate of its trustworthiness beforehand, while the interaction stage facilitates the assessment of trustworthiness during the actual interaction. Within both stages, the performance evaluation of three key components is undertaken, perception, motion and grasping, and speech.

**Fig. 12:**
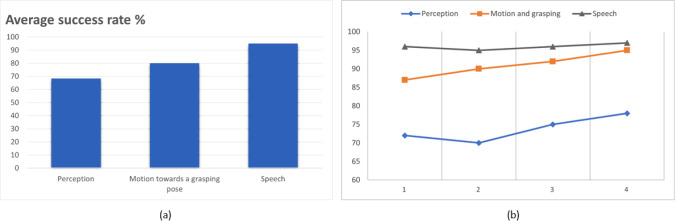
The results of the robot’s trustworthiness about its components. **a** is the measurement beforehand, and **b** is the measurement during interaction

***Self-estimation measure: Build trustworthiness beforehand***

The acquisition of experience by the robot occurs autonomously, without the need for human observation. This implies the incorporation of diverse methodologies to facilitate this process. For instance, in the context of motion towards grasping, a specific function has been implemented to compare the gripper state of the robot with the extracted features of the target object. If the comparison yields satisfactory results, the trial is recorded in the database as a successful instance (The same strategy has been applied for the perception and speech). This stored information is subsequently utilised for the evaluation of trustworthiness during interaction, as elaborated in the subsequent paragraph.

Figure 12a presents the outcomes pertaining to the robot’s performance in the preliminary stage. The average success rate has been computed for each component. Notably, the presence of noise resulting from illumination has an impact on the extracted features derived from the perception system. Nevertheless, it can still be deemed reliable when perceiving the human state. Conversely, most failures in perception occur in the domain of object detection, which consequently affects the manipulation of the identified object. To address these challenges, a series of test scenarios has been proposed, encompassing motion and grasping tasks in both cluttered environments and scenarios involving a single object.

***Self-estimation measure: Build trustworthiness during interaction***

Figure 12b presents the outcomes pertaining to the robot’s performance during interactions. In order to assess the performance of each component in intermittent trials conducted on different days with varying light conditions, four distinct groups were established and categorised. The evaluation of the robot’s performance during interactions was conducted using the following equation.

$$\begin{aligned} D = \sum \frac{d_s}{d_t} \end{aligned}$$

where, $d_s$ represents the number of successful trials, and $d_t$ denotes the total number of trials.

Notably, the speech component exhibits a remarkable level of stability, while the motion and grasping components demonstrate progressive improvements. This improvement can be attributed to the incorporation of a pre-grasping pose, which prompts the motion planner to generate a feasible and optimal trajectory within the robot’s joint space towards the desired goal. The performance of the perception component varies across the four groups, particularly in the second group where the presence of noisy lighting conditions adversely affected object detection accuracy. This information is utilised during each interaction, wherein the robot assesses its own performance and subsequently informs the human counterpart regarding the current status. The human user is then given the opportunity to accept or reject this information and make informed decisions accordingly.

## Discussion

### Scalability and Responsiveness

The proposed framework is specifically designed to cater to the research community and is intended for utilisation in shared tasks involving multi-agents/systems within the robotics domain, wherein humans are also involved in the action loop. In order to evaluate the system’s scalability and responsiveness, we conducted assessments regarding the number of episodes stored in the dynamic memory, as well as the count of relations/axioms within the ontology (static memory). Through multiple experimental runs, we have accumulated approximately 850,000 interactions within the dynamic memory at the time of writing this article. Remarkably, this only accounts for around 2% of the total memory capacity (32 TB). The reasoning process performed on this memory exhibits an average time of 0.85 s.

**Fig. 13:**
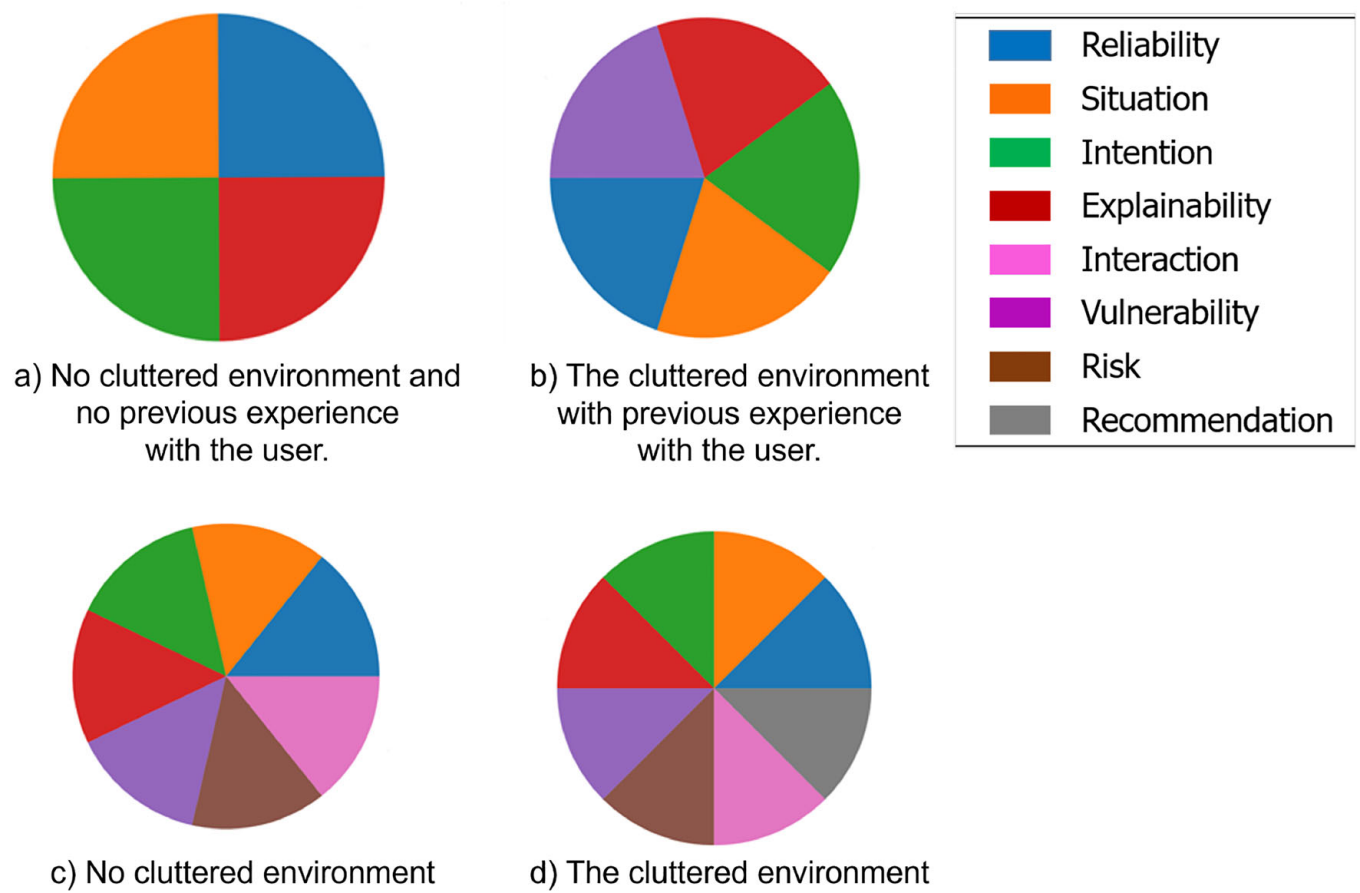
Factors affecting trust measurements in the proposed scenarios. The first row **a** and **b** shows the factors utilised for trust evaluation in the coffee preparation task. The second row **c** and **d** shows the factors utilised for trust evaluation in the cutting fruits task. Our hypothesis suggests that all trust factors currently hold equal weight. In future work, we aim to personalize these weights based on user characteristics like culture and gender

Regarding the static memory, which comprises the ontology knowledge database, encompassing three ontological levels, we have a total of 2,217 axioms. Notably, the performance of the built-in reasoners in the Protégé editor, Pellet and HermiT, pales in comparison to the superior performance exhibited by the Konclude reasoner (as presented in Table 3). In our current research, we also employ cutting-edge tools for parsing and reasoning tasks, utilising implemented wrappers such as Neo4j 10 and RDFlib.^11^ These wrappers facilitate various functionalities, including serialisation, supporting ontology exportation formats such as Resource Description Framework (RDF) and Extensible Markup Language (XML). Additionally, we utilise an implicitly object-relational mapper to seamlessly map data from diverse database sources. Furthermore, we have devised a mechanism for updating rules/axioms to ensure the framework remains adaptable and up-to-date.

### Adaptability and Flexibility

The TICK system demonstrates a high degree of flexibility, enabling various customisation options for specific tasks. This flexibility encompasses the ability to incorporate specific sensors required for a particular task, including a robust tools, modify the ontological models to align with task requirements, choose suitable reasoners for online or offline purposes, and integrate domain-specific ontologies by aligning them with the meta-level.

The system demonstrates flexibility in sensor integration by establishing communication channels between sensory sources and the employed database. This enables the seamless addition of required sensors to accommodate task-specific demands. Regarding ontological flexibility, the system exhibits the capability to automatically estimate trust and its associated factors in diverse contexts, as depicted in Fig. 13. This estimation process encompasses scenarios ranging from the introduction of a single unrisky object to complex situations involving risky objects within cluttered environments. Furthermore, the system empowers users to define and describe abstract concepts, including trust, vulnerability, and risk. These concepts enable inference and information retrieval during interactions. A rule-based model is employed to establish relationships between these concepts, facilitating their interpretation as natural language and exportation as a knowledge graph structure. This flexibility allows users to readily modify the meaning and retrieval of information from the comprehensive database stored in TICK, which utilises PostgreSQL as its underlying technology.

From an implementation perspective, the flexibility can be outlined as follows:

**Table 4:** Comparison between our ontology and other ontologies for trust

Ontology	Domain	Real experiment	Upper-level foundation
TICK	HRI	Yes	Dolce+Dul
[33]	HRI	No	UFO
[34]	Multi-agent	No	No
[35]	Semantic web	No	No

**Table 5:** Comparison between the terms proposed in the Tick and other ontologies

Term	Tick	[33]	[34]	[35]
Trust	Yes	Yes	No	No
Trustor	Yes	Yes	Yes	Yes
Trustee	Yes	Yes	Yes	Yes
Task	Yes	No	No	No
Action	Yes	Yes	Yes	Yes
Behaviour	Yes	No	No	No
Capability	Yes	Yes	Yes	No
Interaction	Yes	No	No	No
Situation	Yes	Yes	Yes	No
Risk	Yes	Yes	Yes	No
Event	Yes	No	No	No
Intention	Yes	Yes	No	No
Belief	Yes	Yes	No	No
Preference	Yes	No	No	No
Vulnerability	Yes	Yes	No	No
Goal	Yes	No	No	No
Recommendation	Yes	No	Yes	Yes
Personalisation	Yes	No	No	No
Explainability	Yes	No	No	No
Social attribute	Yes	No	No	No
Threat	Yes	No	No	No

1. Modelling ontology: The system emphasises interoperability by utilising common vocabularies from a cognitive-based upper-level foundation. This approach broadens the system’s scope and facilitates the integration of existing ontologies.
2. Reasoning interface: Two interfaces have been proposed for flexible inference.
  Reasoning using Prolog predicates, similar to existing approaches, allows the robot to query the TICK ontology using SWI-Prolog and its semantic web library. A wrapper has been implemented to ground the reasoned information as ROS services, which the robot can freely access when required. Additionally, wrappers have been developed for popular reasoners such as HermiT, Pellet, and Konclude.
  Reasoning using implemented wrappers such as Neo4j, owlready2, and RDFlib. These wrappers provide serialisation and support for various ontology exportation formats such as RDF and XML. An implicitly object-relational mapper facilitates data mapping from different database sources, and mechanisms for updating rules and axioms are also provided.

Both approaches offer users the flexibility to leverage existing task-specific ontologies that can be integrated into the TICK ontology. Additionally, they provide adaptable reasoning capabilities based on various reasoning frameworks.

### Comparative Analysis of Ontologies for Trust in HRI

In this section, we review frameworks that use ontologies to support trust. We look for projects that fulfill a set of inclusion criteria and compare them with each other with respect to the reasoning scope of their ontology, the cognitive capabilities of ontologies to increase robot intelligence, and the application domain they applied. We follow the inclusion criteria presented in [58].

Table  4 presents an analysis between the trust ontologies, including our proposed TICK ontology. The table aims to provide insights into the domains to which these ontologies are applied and their alignment with upper-level foundations. Notably, we have identified the ontology proposed by Amaral et al. [33] as the most closely related work. This ontology specifically addresses trust and has been aligned with the Unified Foundational Ontology (UFO) 12 Given our research focus on evaluating the efficacy of the proposed framework in real scenarios, we have undertaken additional efforts to ground the ontological information and establish a comprehensive framework that encompasses the reasoning, planning, and sensing capabilities necessary for the HRI domain. These efforts enable the practical implementation and validation of our ontology within real-world contexts, distinguishing our work from others in the field.

Table 5 provides an assessment of trust ontologies, including our TICK ontology. The objective of this table is to identify and examine the specific terms and concepts supported by these ontologies. Notably, the terms proposed in our ontology are utilised for this comparison. Upon analysis, we have identified the work by Amaral et al. [33] as the most comparable in this regard. However, it is important to highlight that our ontology encompasses additional factors such as explainability, recommendation, personalisation, and other relevant concepts. These concepts have been explicitly modeled within our ontology and subsequently applied in real-world scenarios. This extension of our ontology with these key factors sets our work apart and enhances its applicability within practical contexts.

Table 6 presents a particular focus on their reasoning scope. The objective of this table is to ascertain which ontologies or frameworks are capable of supporting the cognitive capabilities of robots. Our analysis reveals that the proposed architecture, TICK, possesses a distinct advantage in this domain, by applying reasoning techniques in real-world scenarios, thereby establishing TICK as a novel, capable ontology in terms of its cognitive capabilities. The ability of TICK to effectively employ reasoning mechanisms sets it apart from other ontologies and enables the realisation of advanced cognitive functionalities in robot systems.

**Table 6:** Comparison between the cognitive capability of TICK and other ontologies

Cognitive capability	TICK	[33]	[34]	[35]
Recognition and categorisation	Yes	No	No	No
Decision making	Yes	No	No	Yes
Perception and situation assessment	Yes	No	Yes	No
Reasoning and belief maintenance	Yes	No	No	No
Adaptability	Yes	No	No	No
Remembering and learning	Yes	No	No	No
Interaction and communication	Yes	Yes	No	No
Execution	Yes	No	No	No
Theory of mind (TOM)	Yes	Yes	Yes	Yes

Table  7 provides insights into the extent to which these criteria are satisfied by each ontology. Similar to the majority of existing approaches, TICK has been implemented using the RDF and OWL. This choice of implementation aligns with prevalent practices in the field, ensuring compatibility and interoperability with other ontological frameworks. Transparency is another notable characteristic of TICK. It is an open-source library that includes comprehensive documentation, allowing researchers and practitioners to access and utilise it freely. This openness promotes transparency, fosters collaboration, and facilitates the reproducibility and further development of research in the domain of trust ontologies.

**Table 7:** Comparison between the inclusion criteria applied to TICK and other ontologies

Inclusion criteria	TICK	[33]	[34]	[35]
Ontology scope	RDF/OWL	OntoUML	RDF/OWL	RDF/OWL
Decision making	Yes	No	No	Yes
Reasoning scope	Yes	No	No	No
Transparency	Yes	Yes	No	No
Accessibility	Yes	Yes	No	No

Our evaluation focused on several criteria, including the concepts defined within the ontologies, their utilisation in supporting the cognitive capabilities of robots, and the specific application domains they targeted. Through this comparison, distinct advantages were identified in our approach:

1. Alignment with an upper-level foundation that possesses cognitive capabilities, fostering a shared understanding of concept meanings among agents. This alignment enhances the sharability and interoperability of our system, facilitating integration with other domain ontologies.
2. Comprehensive representation of trust and its associated factors, capturing the nuances described in existing literature through the formulation of ontological concepts. This provides a symbolic commonsense comprehension of trust phenomena.
3. Development of reasoning capabilities that enable robots to ground stored information in real scenarios, enhancing their ability to make informed decisions and adapt their behaviour based on the situation at hand.
4. Provision of an open-source tool 13 freely available for utilisation by the research community.

In concluding the comparison with other approaches, it is noteworthy to mention that our approach extends beyond existing studies. Specifically, our work considers factors such as the inference of human intention and the influence of personalisation on trust, expanding the scope of the investigation beyond factors like dependability, reliability, predictability, and faith addressed in a related study [59]. By incorporating these additional dimensions, our approach offers a more comprehensive understanding of human trust in the context of human–robot interactions.

## Conclusion and Future Work

The TICK system demonstrates remarkable adaptability in perceiving diverse trust factors within various contextual settings. It is equipped with perception, planning, and reasoning capabilities, that serve to determine trust in the context of HRI. The framework encompasses past knowledge and situation awareness, which enable the assessment of trust. Personalisation opportunities are identified in both sensor data interpretation, through trust estimation, and behaviour generation. The utilisation of interaction memories and multi-level ontologies allows reasoning about the optimal course of action based on prior interaction outcomes with specific users. The algorithms presented in this work are exemplified through a prototypical task involving object handover from a robot to a human. Nonetheless, the overall system design and its components are intended to be general and applicable to other domains. The separation of ontologies into domain-agnostic and domain-specific components facilitates modular modifications for new domains, while the perceptual algorithms are designed to adapt to different types of sensor data features.

Future efforts will concentrate on enhancing the robot’s performance during interactions with humans. This will involve two primary objectives: (a) improving the system’s ability to perceive and reason about different types of trust such as moral trust [60, 61], aiming to enhance the adaptability and personalisation of the trust model, and (b) improving the quality of feature extraction from the perception modules by employing fusion techniques. Additionally, efforts will be directed towards enhancing the adaptability of the framework to encompass different types of trust across diverse domains and among participants from various genders, cultures, and backgrounds. This includes exploring how individuals who were not involved in the system’s development perceive the outcomes of trust inference performance.

## Definitions

### Notions Inherited from DUL: Reproduced Here for Completeness

The following notions are used in our menta-ontology, replicated from standard DUL definitions, and repeated here for completeness, (to check the full definitions mentioned below, please check this link (https://www.w3.org/2005/Incubator/ssn/wiki/DUL_ssn.)

- Information object: *A piece of information, such as a musical composition, a text, a word, a picture, independently from how it is concretely realized* [39].
- Physical object: *Any Object that has a proper space region. The prototypical physical object has also an associated mass, but the nature of its mass can greatly vary based on the status of the object (scientifically measured, subjectively possible)* [39].
- Information realization: *A concrete realization of an InformationObject, e.g. the written document (object) containing the text of a law, a poetry reading (event), the dark timbre (quality) of a sound (event) in the execution (event) of a musical composition, realizing a ’misterioso’ tempo indication.* [39]
- Quality: *Any aspect of an Entity (but not a part of it), which cannot exist without that Entity* [39].
- Event: *Any physical, social, or mental process, event, or state. More theoretically, events can be classified in different ways, possibly based on ’aspect’ (e.g. stative, continuous, accomplishment, achievement, etc.), on ’agentivity’ (e.g. intentional, natural, etc.), or on ’typical participants’ (e.g. human, physical, abstract, food, etc.)*  [39].
- Event type: *A Concept that classifies an Event. An event type describes how an Event should be interpreted, executed, expected, seen, etc., according to the Description that the EventType isDefinedIn (or used in)* [39].
- Task: *An EventType that classifies an Action to be executed. For example, reaching a destination is a task that can be executed by performing certain actions, e.g. driving a car, buying a train ticket, etc. The actions to execute a task can also be organized according to a Plan that is not the same as the one that defines the task (if any). For example, reaching a destination could be defined by a plan to get on holiday, while the plan to execute the task can consist of putting some travels into a sequence* [39].
- Situation: *A view, consistent with (’satisfying’) a Description, on a set of entities. It can also be seen as a ’relational context’ created by an observer on the basis of a ’frame’ (i.e. a Description)*  [39].
- Action: *An Event with at least one Agent that isParticipantIn it, and that executes a Task that typically isDefinedIn a Plan, Workflow, Project, etc.* [39].
- Social attribute * Any Region in a dimensional space that is used to represent some characteristic of a SocialObject, e.g. judgment values, social scalars, statistical attributes over a collection of entities, etc.*  [39]
- Description: *A Description is a SocialObject that represents a conceptualisation. It can be thought also as a ’descriptive context’ that uses or defines concepts in order to create a view of a ’relational context’ (cf. Situation) out of a set of data or observations* [39].

### Additional Notions

In addition to the standard ontological notions that we based on DUL in the previous section, we have added the following ((some definitions are inspired by the American Psychological Association, to check the full definitions, please check this link https://www.apa.org/)

- Trust: * is the process that represents the state of the entities, including agents sharing a task with individuals to achieve their goals, in a series of events that can be calibrated over time in a situation characterized by uncertainty related to agents (e.g., robots) including individuals (e.g., human), or environmental entities*.
- Goal: *is the description of the final state, in the future, of a plan that is required by an agent to execute.*
- Belief *is information about environmental entities, including agents (i.e., humans and robots), in an event that is associated with some characteristic or attribute, that results in gaining faith or confidence in something.*  [62]
- Intention: * for shared tasks, is a description of how to achieve a goal (defined by the task instructions) in a certain task, based on the belief of an agent towards other(s).*
- Threat: * is an event that contains objects that may cause damage or danger, such as a robot picking up a dangerous object (Sharp-edged object like scissors). Moreover, it may lead to the agent’s vulnerability.*
- Vulnerability: * refers to a characteristic of the performance of a system or agent, which signifies its susceptibility to weakness, encompassing both its constituent elements and the interconnections among them, thereby rendering it susceptible to exploitation by an imminent threat event.*
- Risk: * is a probabilistic value that can potentially lead to harm as a result of a Threat event exploiting Vulnerability.*
- Interaction: *Given an event, the interaction among (some or all) the objects/agents participating in it, is the change of the objects’/agents’ qualities and their relationships during the event*  [43].
- Reliability: *denotes the capacity of agents or systems to effectively carry out actions or tasks within a specific situation, thereby instilling confidence in their consistent and dependable performance.*
- Explainability: *is the description of what an agent does or thinks to do.*

## Footnotes

1 A simple version of the definition is—Trust is the belief that someone will assist you in accomplishing your objectives, even when circumstances are uncertain and you may feel vulnerable.

2 A simple version of the definition is—The process, which involves how different groups collaborate and agents aid individuals in achieving their goals, encompasses a series of events that may evolve over time, particularly in situations where uncertainty exists concerning humans, robots, or the environment.

3 In the subsequent description, the notation “dul.” followed by “x” denotes the utilisation of concepts derived from the DUL upper-level ontology, with “x” representing the specific concept under consideration.

4 https://www.imperial.ac.uk/personal-robotics/

5 https://spacy.io/.

6 (https://github.com/IntelligentRoboticsLabs/gb_visual_detection_3d).

7 (http://protege.stanford.edu/).

8 https://www.ros.org.

9 The success rate in this context refers to the robot’s ability to accurately infer human trust and subsequently adapt its behaviour appropriately toward a specific user. To evaluate this, a standard question of “Did you trust my behaviour?” is employed as a means of verifying the accuracy of the robot’s assessment. The question is posed to the user via the speech recognition system at the conclusion of the experiment.

10 https://neo4j.com/.

11 https://rdflib.dev/.

12 https://ontouml.readthedocs.io/en/latest/intro/ufo.html.

13 https://github.com/MohammedDiab1/Trust-ontology.
